# Thin-Film Encapsulation for OLEDs and Its Advances: Toward Engineering

**DOI:** 10.3390/ma18133175

**Published:** 2025-07-04

**Authors:** Songju Li, Linfeng Lan, Min Li, Zhuo Gao, Xiaolin Yan, Dong Fu, Xianwen Sun

**Affiliations:** 1 State Key Laboratory of Luminescent Materials and Devices, Institute of Polymer Optoelectronic Materials and Devices, South China University of Technology, Guangzhou 510640, China; 202311084815@mail.scut.edu.cn (S.L.);; 2 Guangdong Juhua Printed Display Technology Co., Ltd., Guangzhou 510700, China; 3 Guangzhou New Vision Optoelectronic Co., Ltd., Guangzhou 510530, China

**Keywords:** thin-film encapsulation, large-size OLEDs, ultra-flexibility, thinning, multifunctionality, engineering

## Abstract

Thin-film encapsulation has been a critical method to realize small-size OLED displays. However, the manufacturing of large-size flexible OLED is still in the preparatory phase prior to commercialization, which entails more rigorous demands for reliability and flexibility with regard to thin-film encapsulation. This review, from the perspective of engineering for mass production, addresses the development of thin-film encapsulation and its three core properties for comprehensive validation while engineering, including basic properties, reliability, and compatibility. Moreover, considering the prospective evolution of display products, the review on novel thin-film encapsulation was conducted to evaluate the potential engineering value for thinning, ultra-flexibility, multifunctionality, novel equipment, and emerging technology. It is anticipated that some of the aforementioned technologies may prove to be of significant engineering value. It is therefore hoped that by comprehensive engineering verification, the commercial application of novel thin-film encapsulation can be promoted and the competitiveness of OLED products can be effectively enhanced.

## 1. Introduction

Since the advent of the first computer, displays have served a crucial role in facilitating information interactions between humans and machines. From the first Cathode Ray Tube displays to Liquid Crystal Displays (LCDs), display technology has evolved over hundreds of years. Today, the Internet of Things technology connects thousands of smart electronic devices that come in all shapes and sizes. However, no matter how these devices change, they are inseparable from human interaction, and display is the most intuitive and convenient form of interaction, so IoT technology puts forward a higher demand for display technology: a better display performance and more diverse display forms. Among them, the ingenious design of IoT devices requires displays to be ultra-thin and light, and the novel appearance requires displays to be deformable and flexible.

Traditional displays, including LCDs, usually offer a straight, regular imagination. For a long time, the square shape has become the standard shape of the display. However, the display technology of Organic Light-Emitting Diodes (OLEDs) has broken such limitations. With their low power consumption, high brightness, high contrast, fast response speed, natural color performance, and flexible display [1,2,3,4,5,6], OLEDs, and more recently, quantum dot OLEDs, have kept pace with LCDs and are expected to replace LCDs as the emerging display technology. Among the advantages, being able to fabricate a flexible display is the most prominent feature of OLEDs. The most basic structure of an OLED device includes a substrate on which the device is formed, the anode that injects holes, the cathode that injects electrons, and the organic light-emitting layer that forms excitons from the injected holes and electrons and emits light. The sandwich structure was first proposed by Adachi et al. [7] in 1988 and further optimized to a five-layer structure [1], and nowadays there are even seven-layer structure devices [8]. The efficiency and lifetime of the devices have been gradually improved and have reached the requirements for commercialization. However, regardless of the changes in the OLED devices themselves, organic materials are naturally less resistant to water vapor/oxygen in the environment than inorganic materials. Some mechanism studies have shown the crystallization and degradation of organic materials and the oxidation of metal electrodes after exposure to water vapor/oxygen [9,10,11]. This ultimately leads to the failure of OLED display devices and the generation of display dark spots. For this reason, it is necessary to encapsulate the OLEDs with the purpose of isolating the device from the external environment and preventing water vapor and oxygen from invading the device and causing failure.

Traditional OLED encapsulation utilizes a sandwich seal in which OLEDs are in the middle of two pieces of glass, one of which is the glass substrate and the other is the cover glass. The glass itself exhibits excellent barrier properties with regard to water vapor. Consequently, a simple encapsulation can be achieved by applying a UV-curable adhesive between the two pieces of glass. More specifically, the UV-curable adhesive is applied in the area around the OLEDs.

However, due to the rigidity of the glass material, the aforementioned encapsulation cannot be used in flexible OLED displays. For realizing flexible displays, in addition to adjusting the glass to an organic flexible substrate, flexible encapsulation technology has become the most important part of the flexible OLED display. Nowadays, thin-film encapsulation (TFE) technology has been the mainstream encapsulation technology to realize flexible OLED encapsulation. By depositing a single or multiple layers of thin film (such as alumina, silicon oxide, silicon nitride film, etc.) on the OLEDs, the OLED displays can be guaranteed to operate normally as well as to achieve a flexible shape. These thin films exhibit a high gas diffusion barrier property, a thin thickness, and a low Young’s modulus.

**Figure 1:**
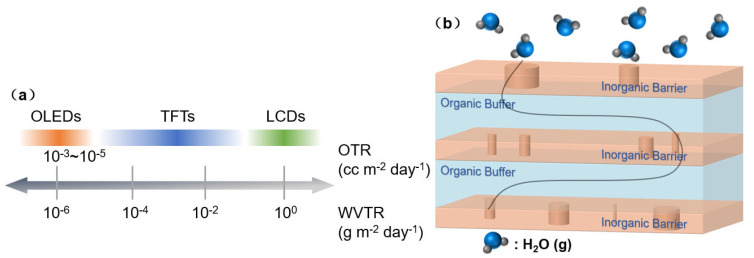
(**a**) Requirement of WVTR for different devices. (**b**) Schematic mechanism for increasing water vapor intrusion pathways.

Gas diffusion barriers (GDBs) were initially developed from food packaging technology to maintain the freshness of food. However, due to the sensitivity of electronic devices to water and oxygen (particularly organic electronic devices), the conventional barrier film employed for food packaging is inadequate for protecting electronic devices. As shown in Figure 1a, for example, LCDs require a Water Vapor Transmission Rate (WVTR) of 1 g m^−2^ day^−1^, while OLEDs require 10^−6^ g m^−2^ day^−1^ (for a device service lifetime of around 10,000 h), a difference of six orders of magnitude. Noteworthily, a low Oxygen Transmission Rate (OTR) is also important for GDBs. However, water vapor generally exhibits a faster permeation rate than oxygen and tends to react with barrier films, which causes corrosion. Moreover, from the view of molecular dynamics, the molecular diameters of H_2_O and O_2_ are compatible, so if the WVTR is satisfactory, the OTR can also meet the requirements. In order to achieve a low WVTR, the industry and researchers have focused their efforts on a number of key areas, including the development of ultra-high barrier materials, the evolution of deposition technology, and the design of composite stacked layers. The Barix encapsulation technology [12] designed by VIREX SYSTEM (Wellesley, MA, USA) is an important foundation for TFE in terms of structural design. The main concept of this technology is to form a structure of inorganic/organic stacked layers. The inorganic layer plays the role of a barrier to water vapor permeation. The organic layer was formed by the flash evaporation technology and originated from liquid-phase organic monomer vapor, which exhibited strong mobility on the depositing surface. The precursor film, prior to curing, was capable of flattening the depositing surface, even when impurities or particles were present, by wrapping them in organic materials. Furthermore, the deposition of an inorganic barrier layer on a flat surface resulted in an enhanced performance. As shown in Figure 1b, the organic layer also blocks the continual growth of micro-defects (e.g., pinholes) in the inorganic layer during the deposition process, so that the defects between the upper and lower inorganic barrier films (through which water vapor/oxygen can easily penetrate) are misaligned with each other, and thus water vapor/oxygen molecules need to take a much longer path to intrude into the encapsulated device. By stacking multiple sets of inorganic/organic films, the WVTR can be effectively reduced.

Nowadays, OLED display technology has been integrated with thin-film transistors (TFTs), which provide a superior driving capability [13,14,15], thereby achieving Active-Matrix Organic Light-Emitting Diodes (AMOLEDs) display technology. This technology has been applied and commercialized in small- and medium-size display products (such as the display of smartphones and smart wearable devices). According to a survey, the flexible AMOLED smartphone panel accounted for 77.8% of the display market in the fourth quarter of 2023, which has long surpassed LCDs to become the main display technology for small- and medium-size display panels in the world, and TFE has also become a mainstream encapsulation technology for OLEDs. In contrast, the application of large-size OLED display products (mainly used in televisions, computer monitors, tablets, etc.) is relatively limited, and the number of shipments is low. This is mainly because the production cost of large-size OLED display panels is very high, and the technologies to achieve a stable yield and high reliability still need detailed study and engineering verification. In consideration of OLED encapsulation technology, it is observed that large-size products often require a longer service lifetime: 10 years for TVs, 5 years for monitors, and 3 years for smartphones. Therefore, TFE must demonstrate enhanced reliability to ensure a longer service lifetime of OLED display products. Furthermore, the advent of IoTs has given rise to a diversification in display formats. In addition to the emergence of foldable displays, there has also been a progression toward rollable and stretchable displays, coupled with a reduction in both the folding and rolling radius. This has necessitated a corresponding enhancement in the flexibility of TFE. Moreover, in order to solve the color crosstalk problem encountered by Micro-OLED displays [16], TFE is required to develop into a thinner thickness (~1 μm). To sum up, the current TFE technology still has major limitations, so it is necessary to continue engineering research and its verification.

The TFE technology for OLEDs has been researched for nearly 20 years, and a variety of films with ultra-high barrier properties have been developed, as well as ultra-flexible structures. However, when these technologies are applied to real flexible OLED displays, it is far from sufficient to achieve barrier properties and flexibility solely, and their reliability, transmittance, particle passivation ability, adhesion, chemical resistance, and material hydrogen content; the cost, efficiency, and equipment for employing these technologies; and their compatibility with OLEDs/TFTs need to be further investigated and verified. Consequently, engineering represents a critical step in the transition of TFE technology from lab to commercialization in order to ensure its full functionality.

The engineering of the product encompasses the entire process, from the initial concept design to the product’s market launch. This process is conducted in accordance with the engineering procedures and specifications, utilizing scientific methods, unified management, and control systems. The objective is to guarantee the quality, manufacturing progress, and costs of the product, thereby achieving the optimal outcome. The authors consider that engineering embodies the concepts of unification, integration, and verification. And, the engineering necessitates many items to be studied regarding TFE: (i) the influence of TFE’s detail process, (ii) the adjusting method of the detail process according to the difference of products, (iii) the optimization of coordination with TFE adjacent layers, (iv) the influence of TFE on OLEDs/TFTs, (v) the cost of TFE (including equipment, materials, and product cycles) and the commercialization route, (vi) the failure modes of TFE, and (viii) the demand for TFE’s emerging technologies for new products.

This paper attempts to review, from the perspective of engineering, the development and application of TFE as well as the research of novel TFE technology. Initially, by understanding the development of OLED encapsulation technology, the advantages and disadvantages of TFE are clarified. Then, this paper summarizes several performance indices required for TFE to function reliably for OLEDs. Subsequently, the research on the barrier principles and failure mechanisms of TFE is reviewed, which provides first principles for the analysis of product failure caused by TFE during the engineering phase. Furthermore, based on the new requirements of new products for TFE, the research progress of novel TFE technology is reviewed from five directions: thinning, ultra-flexibility, multifunctionality, novel equipment, and emerging technology. Finally, the future development of TFE technology is prospected.

## 2. Fundamentals of Thin-Film Encapsulation

### 2.1. Development of OLED Encapsulation Technology

**Figure 2:**
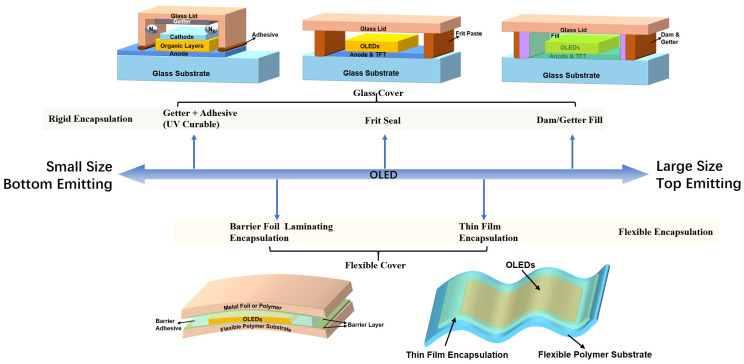
Simple classification and development path of OLED encapsulation technology.

The primary purpose of OLED encapsulation is to protect the whole OLED emitting region against environmental water vapor/oxygen, so encapsulation material is always formed with a whole package shape around the entire OLED pixel array and is a part of the display itself. As shown in Figure 2, initially, OLED encapsulation would use the “cover (Lid)” to wrap the OLEDs, whether the “cover” is rigid or flexible. Therefore, the development of OLED encapsulation technology is inseparable from the development of the OLED product itself. Different from the small to large size, the bottom-emitting to the top-emitting type, the encapsulation methods are different. This section will introduce the desiccant + UV adhesive encapsulation, Frit Seal encapsulation, and dam/fill encapsulation method, which use a rigid “cover” (glass). The lamination encapsulation and TFE method will be introduced subsequently, while these methods utilize a flexible “cover” (barrier film).

Desiccant + UV adhesive encapsulation is the earliest developed OLED encapsulation method [17], which uses glass as a substrate, UV adhesive for the bond of substrate and glass, and OLEDs are placed between them. This encapsulation method could achieve a 40% decrease in luminous brightness compared to the initial brightness of the OLED, even after about 1000 h. Due to the UV adhesive generally not exhibiting the available gas diffusion barrier performance, in order to prevent OLEDs from the side invasion, a desiccant is generally needed to be placed between OLEDs and the cover glass. The above encapsulation method is relatively simple, does not require high control of the production process, does not require high-precision adhesive coating technology, and is suitable for the small-size and pilot-scale production of OLEDs. When using desiccant to increase the encapsulation effect, the encapsulation method can only be applied to bottom-emitting OLED displays (the light emitted by the OLED is emitted from the substrate side, and the display observation surface is on the substrate side) due to the desiccant’s light-blocking effect.

Frit Seal encapsulation uses an adhesive material containing glass microparticles as a frame adhesive to be applied around the OLED display area. After temporarily sealing the two pieces of glass and coating an adhesive frame around OLEDs between the middle of the glasses, laser irradiation is used to ablate the frame adhesive, at which time the glass microparticles inside the frame adhesive will be heated and melted, and the molten frame of glass and upper and lower glass covers would form an integrated whole, so as to realize the lateral encapsulation. This method greatly improves the reliability of OLED products and is the most reliable, low-cost rigid encapsulation method for small- and medium-sized OLEDs. However, due to the particles contained in the frame adhesive, the general needle-type gluing equipment is easy to block, so the frame adhesive is basically completed using the screen-printing process. The screen-printing process is less accurate, the screen maintenance and replacement frequency are high, and inappropriate maintenance or process control would easily lead to the broken-glue or the less-glue phenomenon, thus forming a path of water vapor/oxygen erosion. When expanding this method to the encapsulation of large-size OLEDs, the issues of uniformity, precision, and stability are difficult to ensure.

The encapsulation process of dam/fill encapsulation is as follows: (i) coat a frame of dam material at the glass cover on the side toward the OLEDs; (ii) spray the fill material inside the frame of the dam; (iii) laminate the glass cover containing the dam/fill with the glass substrate carrying OLEDs; and (iv) cure the dam/fill with ultraviolet or heat radiation to bond the two substrates on both sides. This type of encapsulation has higher material requirements [18], which on the one hand requires that the framing adhesive should have a certain gas barrier ability, so that it can block the side intrusion of water vapor to a greater extent. Because the frame glue is organic, the barrier ability is limited, so an additional frame of adhesive with getter material can also be formed aside from the frame of the dam. The getter can absorb the water vapor from side intrusion, which can greatly improve the reliability of encapsulation. Similarly, water vapor can be further absorbed by adding desiccant components to the fill material. Tsuruoka et al. [19] produced a 3.5-inch OLED full-color display by adding desiccant containing aluminum complexes to the fill adhesive, which can consume water vapor by reacting with it to form an organic hydroxide, and finally improved the water vapor barrier performance. On another hand, the fill adhesive is required to have great spread ability on the glass substrate, and it itself needs to have a good leveling performance so that even if the surface of the OLED display to be encapsulated is uneven, it can still fill the gap between the OLED and the encapsulation cover. This is one of the positive characteristics of this encapsulation method: due to the presence of fill adhesive, even if the size of the substrate and cover becomes larger, it will not exhibit a MURA (a word originated from Japanese, which means nonuniformity, speckle, and instability) phenomenon such as Newton’s Ring. Newton’s Ring, which deteriorates the display performance, is usually caused by the uneven height of the voids between the substrate and cover. Therefore, this encapsulation method is the preferred solution for large-size rigid OLED encapsulation. What is more, for top-emitting OLED devices, when the outgoing light is emitted from the top of the OLEDs, if there is no filler adhesive, the outgoing light would first pass through the air between the two substrates to enter the cover glass and finally enter the air again from the cover glass. Due to the high refractive index of OLED material (1.8–2.1), the total reflection angle is small, making it easier for light emitted from OLEDs into air (refractive index = 1) to be reflected. This results in some of the emitted light being lost. The light then passes through the glass cover with a refractive index of 1.45 and then enters the air again, and the outgoing light will be lost again. In this case, by filling the space between the OLED and the cover glass with a transparent organic material (refractive index of about 1.5) that can buffer the outgoing light, the light extraction efficiency of the OLED can be effectively improved [20].

In fact, the above rigid encapsulation methods, by thinning the glass substrate or replacing it with ultra-thin glass/metal sheets, can also be utilized in flexible displays to a certain extent. However, this kind of display is often limited in terms of its flexibility, which is only suitable for products featuring slightly curved screens or similar. This could not be considered truly flexible. To achieve a truly flexible display, any encapsulation materials featuring high hardness and low flexibility must be replaced. Among them, the rigid glass substrate and cover, UV adhesive, and frame of the dam should be removed. For example, in terms of the dam/fill encapsulation, the fill adhesive should offer high water vapor barrier performance after the frame adhesive has been removed. And after the removal of the glass substrate, it is required to utilize a flexible substrate with enough barrier properties. The lamination encapsulation with barrier film can meet the above requirements. A feature of this method is that the flexible substrate has high barrier properties, and when the OLED is the bottom-emitting device, metal aluminum foil with high gas diffusion barrier properties can be used as the flexible “cover”. When the OLED is a top-emitting device, a transparent organic flexible substrate coated with a gas barrier film should be used as the “cover”. For details of the process flow, the flexible “cover plate” and the flexible substrate carrying OLEDs and other functional devices are laminated together by using a roller or a drum to complete the encapsulation. To ensure the two substrates bond together and prevent lateral water vapor intrusion, a high-specification resin-based bonding layer is required: strong bonding strength, gas diffusion barrier ability, temperature stability, flexibility, light transparency, and low haze. The gas diffusion barrier can be realized in terms of both hydrophobicity and barrier properties [18]. Hydrophobicity can be achieved by using hydrophobic resins and adding inorganic fillers, while barrier properties can be achieved by increasing crosslink density, thermal stability, and adding anisotropic fillers (increasing water vapor/ oxygen intrusion paths).

Barrier film laminating encapsulation can be easily fabricated, requiring low equipment costs. However, as the requirements for the barrier film and resin are extremely high, the cost of the materials is also very high, which limits the application of this method; thus, the cost of material is very high, which limits the application of this method to a certain extent. At the same time, the overall thickness of the encapsulation structure is bound to be at least 100 μm, which contains a barrier film, barrier resin, and flexible “cover”, which could not meet the demand for high flexibility to achieve an ultra-small bending radius of the flexible display. At this point, the advantages of thin-film encapsulation come to the fore. By directly depositing thin films with high gas barrier properties on OLEDs and stacking them into a multiple-layer structure, such as an inorganic–organic–inorganic encapsulation structure, it is possible to effectively increase the path of water vapor/oxygen intrusion and improve gas diffusion barrier properties. Moreover, the TFE’s properties of the film’s conformality (which can wrap the OLEDs), strong adhesion, and thin thickness have promoted it to be the preferred encapsulation solution for flexible OLED displays. More TFE’s features will be described in Section 2.2.

### 2.2. Functioning of Thin-Film Encapsulation

**Figure 3:**
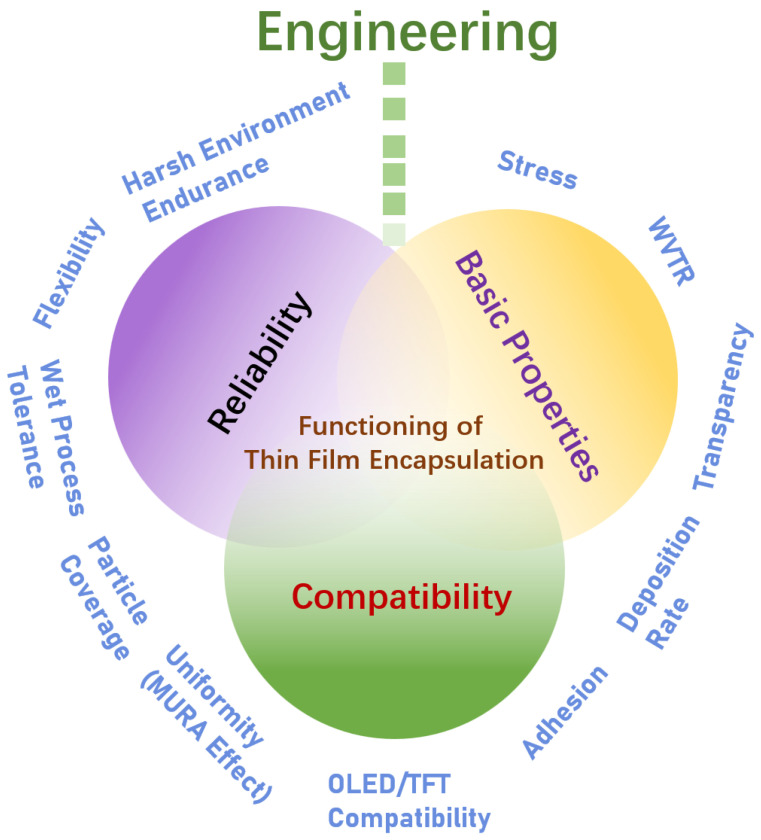
Items of property for the complete functioning of TFE while engineering.

Thin-film encapsulation technology serves to protect OLED displays from the intrusion of water vapor/oxygen into the display pixels. This is achieved by depositing thin films around the display area to isolate external gases. Consequently, the gas diffusion barrier property represents the most fundamental performance of TFE. This is typically quantified by the WVTR. However, as a display encapsulation technology, TFE applied to OLEDs is not only required to exhibit an excellent WVTR value, but also to meet a number of other performance criteria in order to ensure real functionality so that it can exhibit a great display performance and serve for a long lifetime. As illustrated in Figure 3, this paper presents a summary of the essential properties of TFE that are necessary to satisfy for its application to OLEDs, particularly during the engineering phase. In essence, three principal properties can be identified: basic properties, reliability, and compatibility.

#### 2.2.1. Basic Properties

Among the basic properties, in addition to the WVTR, intrinsic stress is also one of the basic properties of the TFE layer of primary concern. This is because, on the one hand, the TFE layer usually seals the entire film, covering the entire display area. If the internal stress of a large-area thin film is too great, the upper and lower films may peel away from each other and the film itself may crack and swell [21,22]. On the other hand, large stress can also easily make the whole display body curve, leading to alignment errors and vacuum adsorption abnormalities while in the manufacturing process.

**Figure 4:**
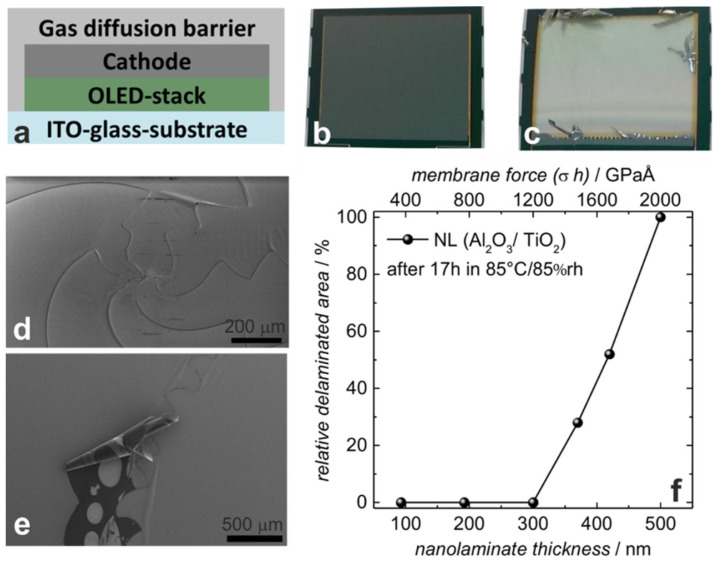
(**a**–**f**) Peeling phenomenon caused by large stress (copyright © 2016, American Chemical Society).

Behrendt et al. [23] developed atomic layer deposition (ALD) Al_2_O_3_ films, TiO_2_ films, and their nanolaminate layers, which all exhibited internal stresses above 400 MPa. The authors encapsulated an OLED display of about 11.5 cm^2^ using the nanolaminate layers and stored the display in a high-temperature and high-humidity environment for a period of time (70 °C/70% RH for 7 days or 85 °C/85% RH for 17 h). The results demonstrated that after high-temperature and high-humidity storage, a notable peeling phenomenon was observed (Figure 4c–e), with the extent of peeling increasing in proportion to the thickness of the TFE layer (Figure 4f). This phenomenon may be attributed to the presence of tensile stress within the internal structure of the film. Furthermore, the impact of high temperature and high humidity resulted in a mismatch of thermal stress between the inorganic encapsulation film and the organic OLED film, due to the difference in thermal expansion coefficients. Ultimately, when the stress exceeds the peeling force between the two films, separation occurs at the weakest point. The weakest point is typically the area around particles (foreign objects such as dust that accidentally deposit on the films), which is generated from the manufacturing environment and process [24]. The peeled film may also buckle or even curl, which is a manifestation of internal stress release [25]. Using this phenomenon, it is even possible to create some micro-three-dimensional structures [25].

**Figure 5:**
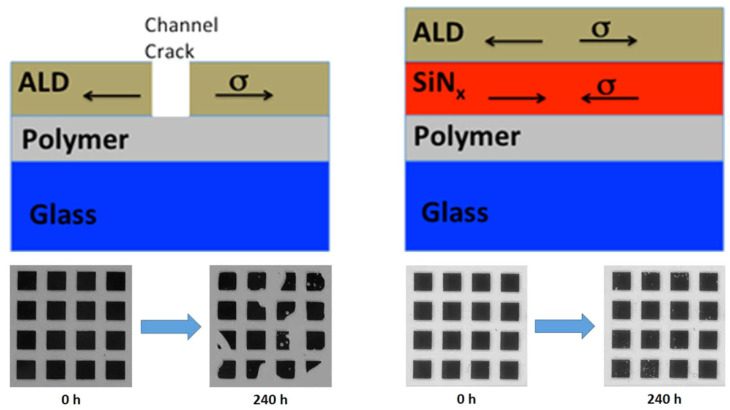
Schematic diagram of the principle of counteracting the opposite-direction stress and its resultant figure.

In order to reduce the impact of the internal stress of the film, the most direct method of obtaining low-stress films is through the development of new materials and processes. Profijt et al. [26] established a bias power supply based on a standard plasma-assisted atomic layer deposition technique. The deposition equipment was used to control the bias voltage, which in turn affected the strength of the plasma bombardment and thus the density of the deposited film and other properties. This allowed the internal stresses of the atomic layer deposited Al_2_O_3_ film to be adjusted from tensile stress to compressive stress. Furthermore, the introduction of films with opposite stress directions to form stacked films is a common method for reducing stress. Bulusu et al. [24] achieved a notable reduction in the failure rate of encapsulated films subjected to high-temperature and high-humidity environments by incorporating SiN_x_ films (performing compressive stresses) between ALD films (exhibiting substantial tensile stresses) and organic layers. As illustrated in Figure 5, the failure rate of encapsulated films can be reduced by the incorporation of stacked films with opposing stress properties. The introduction of a stacked layer with opposing stress properties can effectively counteract the effect of stress and achieve a balanced internal stress level within the overall structure.

**Figure 6:**
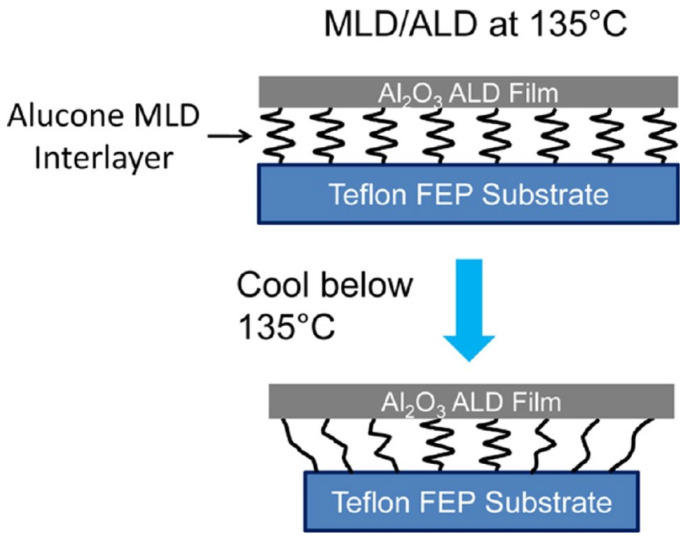
Schematic illustration of the principle of MLD organic layers for buffering thermal expansion coefficient mismatches (copyright © 2013, American Chemical Society).

Another useful method for mitigating the impact of stress is to construct a stack comprising organic films with a low Young’s modulus (i.e., a soft material). To address the issue of the swelling and fracturing of ALD-Al_2_O_3_ films deposited on Teflon organic substrates, a Molecular Layer Deposition (MLD) organic film was interposed between the ALD film and the substrate [27]. The results demonstrated a notable reduction in the incidence of the undesirable phenomena. It is postulated that the rationale behind the efficacy of this methodology lies in the fact that the organic long chains of the MLD films serve to mitigate the thermal expansion coefficient disparity between the substrate and the ALD film (refer to Figure 6).

Transparency represents another fundamental property of TFE films that is crucial to their functionality. This property requires that TFE films exhibit an enhanced light transmittance rate within the visible light spectrum. Differing from a bottom-emissive device, as for a top-emissive device, the light emitted from the OLEDs passes through the TFE. An excessive absorption coefficient may result in a deterioration in the light efficiency of the OLEDs. The most effective method for enhancing transmittance is through the careful selection of materials and processes. For example, the transmittance of a TiO_2_ film may be relatively low; however, the incorporation of a nanolaminate layer comprising Al_2_O_3_ and TiO_2_ can significantly improve the overall transmittance [28]. As an additional example, the O content of SiO_x_N_y_ encapsulated films can be regulated by the process to yield high-transmittance films [29]. It is also necessary to consider the transmittance of organic buffer layers in TFE within the blue light region. As some organic films exhibit significant absorption in the near-UV region, the blue light region may also be influenced by the Gaussian distribution of the absorption versus wavelength. Consequently, this may result in degradation in transmittance within the blue light.

#### 2.2.2. Reliability (RA)

Reliability is the highest quality requirement for OLED display encapsulation. As a reliability evaluating method, the harsh environment endurance test refers to the method to test whether the display still works properly after placing the OLED display in an environment at high temperature and high humidity, or high- and low-temperature cycling for a period of time. Among them, the storage stability in an 85 °C/85% R.H. environment is the most stringent evaluation method for encapsulation. Generally, the reliability requirement of OLED displays is that they cannot produce any display defects after 240~1000 h of storage in an 85 °C/85% RH environment [30]. The purpose of high-temperature and high-humidity storage tests is to simulate the actual use environment of the display on the one hand, and on the other hand, it is a kind of accelerated aging test. Refer to the Arrhenius temperature accelerating factor calculation Formula (1) [31]:

(1) $$T_{AF}=\frac{L_{normal}}{L_{stress}}=exp\left[\frac{E_{a}}{k}\times \left(\frac{1}{T_{normal}}-\frac{1}{T_{stress}}\right)\right]$$

where *T*_*AF*_ is the acceleration factor and *L*_*normal*_ is the lifetime at room temperature (the normal environment), and *L*_*stress*_ is the lifetime at high temperature (the environment where the acceleration stress test is conducted). *T*_*normal*_ is the room temperature, and *T*_*stress*_ is the high temperature (all temperature values are calculated with the unit of Kelvins). *E*_*a*_ is the reaction activation energy, which is generally 0.6 eV for electronic products, but may vary depending on the product. k is Boltzmann’s constant, i.e., 8.62 × 10^−5^ eV/K.

The above Formula (1) only considers the accelerating aging effect by temperature, Hallberg, and Peck combined with the influence of humidity to obtain the acceleration factor (*TH*_*AF*_) under a high-temperature and high-humidity environment (e.g., 60 °C, 90% R.H.). Formula (2) can be utilized [32]:

(2) $${TH}_{AF}=\frac{L_{normal}}{L_{stress}}={\left(\frac{{RH}_{Stress}}{{RH}_{Normal}}\right)}^{n}\times exp\left[\frac{E_{a}}{k}\times \left(\frac{1}{T_{normal}}-\frac{1}{T_{stress}}\right)\right]$$

where *RH*_*Normal*_ is the relative humidity under the regular ambient, *RH*_*Stress*_ is the relative humidity in the test environment, and *n* is an index reflecting the degree of humidity impact, generally 2~3. If the device is utilized in an environment of 25 °C/60% R.H., *n* is set to two (small impact). The accelerated test at 85 °C/85% R.H. for approximately 860 h is equivalent to approximately 10 years of regular use, which is consistent with the estimated product lifetime for large-sized display devices, such as televisions. Furthermore, if a small-sized display product (such as a smart portable device) is replaced every five years, it would be necessary to test for approximately 430 h to confirm whether the display product meets the requisite lifetime requirements.

In addition to being a method for accelerating aging tests, high-temperature and high-humidity environments may also lead to material degradation and mismatches within the encapsulation films readily. Most of the aforementioned peeling phenomena due to excessive internal stresses occur only after exposure in such harsh environments. Guo et al. [33] attempted to improve the encapsulation barrier performance by introducing ALD-Al_2_O_3_ into the full PECVD prepared inorganic–organic–inorganic TFE structure, forming a structure of SiN_x_/pp-HMDSO/Al_2_O_3_/SiN_x_. Although the WVTR can be improved by one order of magnitude, after the RA test, the OLEDs with this structure failed faster than the normal ones. A SEM analysis revealed that the reason was the peeling-off phenomenon between Al_2_O_3_ and the pp-HMDSO (plasma polymer hexamethyl disiloxane) film.

The evaluation of degradation under harsh environments is also a very important part of encapsulation film development. Sun et al. [34] found that some PECVD-prepared SiN_x_ films would be oxidized and transformed into silicon oxide films after storage in harsh environments, which made it easier for water vapor to intrude into OLEDs. ALD-Al_2_O_3_ is a new type of encapsulation material with a high gas diffusion barrier performance; however, when ALD-Al_2_O_3_ is placed in a harsh environment, Al_2_O_3_ is prone to a hydrolysis reaction with water vapor to form the hydroxide of Al [35], and such material transformations lead the originally dense film to become fluffy, forming a large number of water vapor intrusion channels.

In the context of flexible OLED display products, the mechanical reliability of the OLED is a crucial factor in assessing product quality. The current commercial OLED dynamic flexible display products are primarily foldable smartphones, which are capable of displaying a normal image even after being folded at least 100,000 times. This places significant demands on the flexibility of TFE. From a cross-sectional view of the stress distribution across each film, the bending of the OLED display results in the formation of one or more neutral planes [36,37,38,39,40], whereby the film within the neutral plane is subjected to a stress level of zero. Furthermore, the film that is positioned farther from the neutral plane is subjected to an increasingly higher degree of stress as the distance from the neutral plane is increased. The TFE structure may be subjected to two distinct stress states, depending on the folding direction and the distance from the neutral plane. On the convex surface, tensile stress will be present, while on the concave surface, compressive stress shall prevail [41]. A critical stress/strain threshold exists for most plastic materials. For instance, a 20 nm thick ALD-Al_2_O_3_ layer can withstand a critical strain of approximately 1.19 ± 0.22%, which is roughly equivalent to a bending radius of 5.25 mm [42] (most metal oxides exhibit a critical strain of approximately 1% [43]). When a film with limited flexibility is subjected to an externally applied tensile stress which exceeds its critical stress, the film will invariably split and form cracks. Compressive stress tends to cause the film to bulge, which can subsequently lead to peeling and the formation of cracks [44]. The generation of cracks or bulges in the TFE may permit the ingress of water vapor, which could ultimately result in the failure of the OLED.

**Figure 7:**
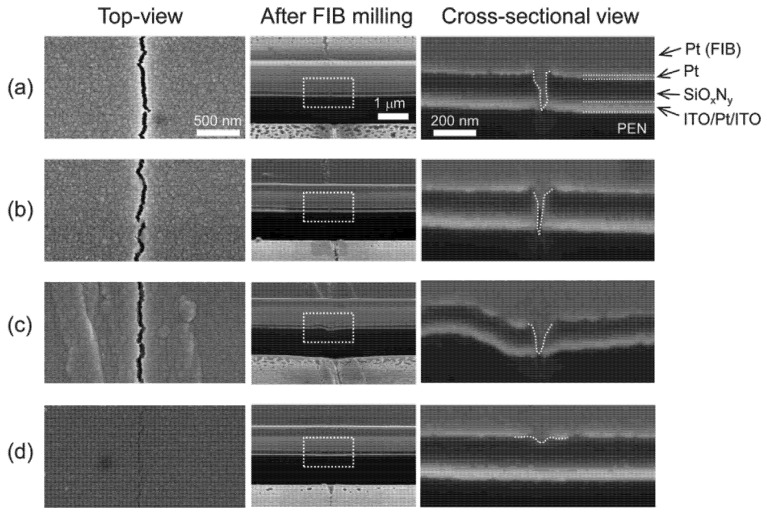
SEM schematics of SiO_x_N_y_ films with oxygen compositions of (**a**) 0.08; (**b**) 0.10; (**c**) 0.47; and (**d**) 1.13, respectively, that produced cracks after bending. The left column of images shows the top view, and the middle and right columns of images show the cross-sectional view and its enlarged view (copyright © 2016, Elsevier B.V. All rights reserved.).

From the perspective of a single-layer film, the flexibility of the film is generally related to the Young’s modulus of the material itself. This is because a smaller Young’s modulus results in a smaller stress endured [36] and a higher critical stress. Consequently, the selection of materials and process optimization represent effective methods for enhancing the flexibility of TFE by reducing the Young’s modulus of the deposited film. As illustrated in Figure 7, Park et al. [45] developed films that can passivate cracks by modulating the oxygen composition of SiO_x_N_y_ deposited by PECVD. Oh et al. [46] conducted a comprehensive investigation on the mechanical reliability of SiN_x_ films under varying stress states, modifying the conditions of PECVD to assess the impact of compressive stresses on critical stress. However, given the lack of film materials that satisfy both water vapor barrier ability and low Young’s modulus, it is challenging to meet the demand for high flexibility by modifying a single film. In response to this challenge, scholars have developed innovative strategies, including the thinning and nanolaminating of TFE films [47], which will be discussed more extensively in subsequent sections.

**Figure 8:**
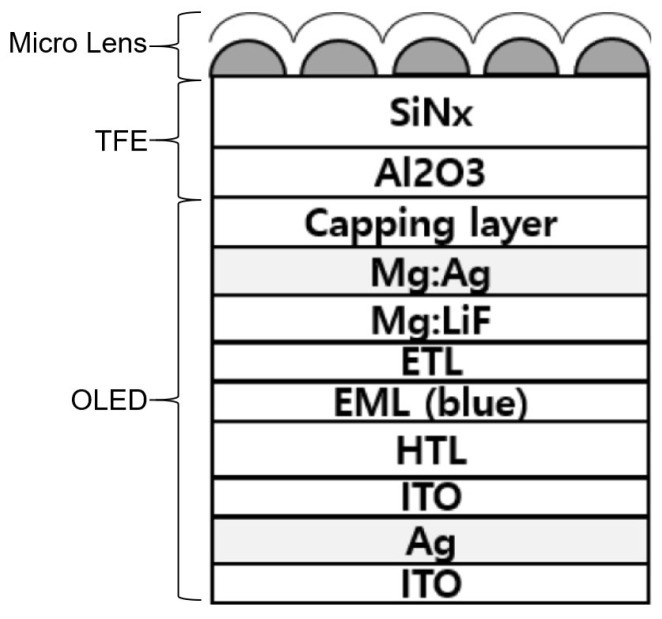
Schematic structure of a microlens on TFE.

**Figure 9:**
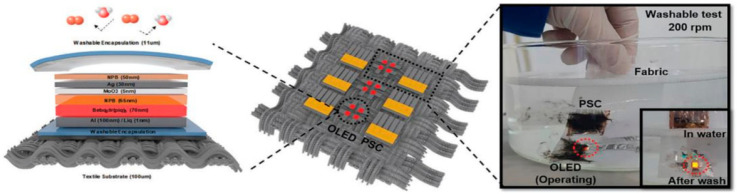
Schematic structure of a fabric display.

In the case of high-value-added OLED display products, some wet processes may be carried out on the top of TFEs [48], including the production of micro lens structures on TFE [49,50,51,52,53], as illustrated in Figure 8. The fabrication of micro lenses typically necessitates a photolithography process, which utilizes photoresist solvents, developers, and deionized water for the cleaning procedure. These materials will inevitably come into contact with the TFE films. It is therefore necessary for the encapsulation film to possess a high level of tolerance to wet processing (wet process tolerance). Moreover, when OLEDs are utilized in fabric displays, as illustrated in Figure 9 [54], the TFE must undergo a multitude of cleaning processes, including water cleaning or chemical dry cleaning. Similarly, the enhancement of the TFE’s ability to withstand long-term exposure to water and chemical solutions necessitates a more rigorous approach to structural design and material configuration. This topic will be discussed in further detail in subsequent sections.

**Figure 10:**
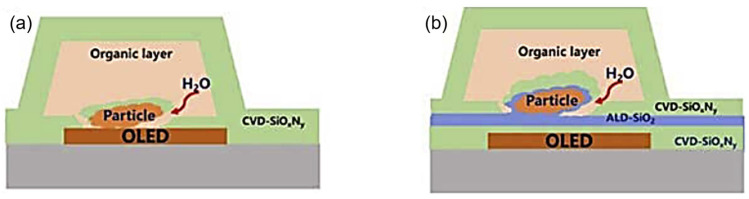
Schematic representation of (**a**) the effect of particles on TFE and (**b**) the role of ALD-SiO_2_ used to cover the particles.

In considering the reliability of OLED displays utilizing TFE, it is imperative to acknowledge the capability to cover particles (particle coverage). In the manufacturing process of OLEDs, it is unavoidable that particles will fall on the display panel during transportation or fabrication. These particles may originate from external sources, such as dust in the environment, or from the deposition process itself, where impurity particles may be generated. Such particles are often statistically significant and difficult to completely eliminate. Unfortunately, TFEs are particularly susceptible in the presence of particles. In most cases, inorganic films on particles are thinner or even disconnected at the particle–substrate contact interface, rendering them unable to form a continuous film (Figure 10a [21]). This results in the ingress of water vapor through these defects into the OLEDs. The conventional TFE structure employs a thick organic film as a particle coverage and planarization layer, which could significantly enhance the reliability of TFEs. This is due to the fact that even if the first inorganic layer presents pathways for water vapor intrusion, the second inorganic layer would not be susceptible to particulate influence thanks to the planarization layer.

However, as the operational lifetime of large-size OLED displays increases, and the operational lifetime of the TFEs also increases, the areas where particles are present are susceptible to becoming weak points that may result in failure after prolonged usage. Furthermore, due to the fact that OLED display panels are usually manufactured with multiple panels simultaneously on a larger substrate during mass production, large-size display panels, which occupy a larger area, are forced to be produced in a smaller quantity on a given substrate. If the same quantity of particles is randomly distributed on a substrate, the probability of a large-size OLED display panel being free of particles is significantly lower than that of a small-size panel. Hence, the yield is reduced.

In addition, the process of using flexible OLED displays increases the probability of films being subjected to stress and consequently generating cracks. This can be attributed to the potential compression of films by adjacent particles when displays undergo deformation (e.g., bending, rolling, or stretching) [39]. It can be inferred that enhancing the passivation ability of TFE to particles and extending the encapsulation lifetime in the presence of particles will be a significant development direction for improving product quality and reducing manufacturing costs in the context of large-size flexible OLED products.

**Figure 11:**
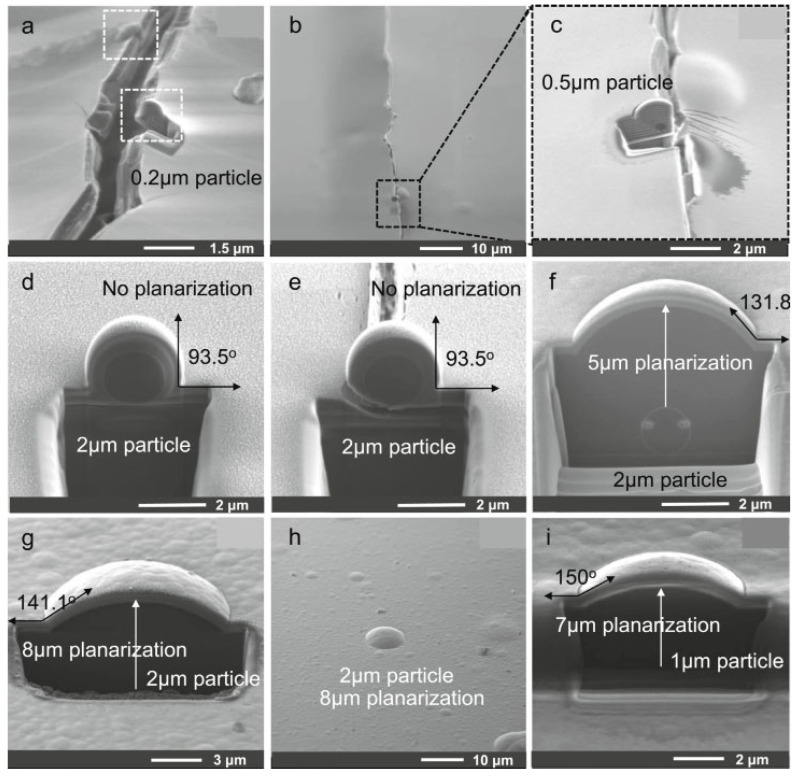
Cross-sectional SEM images of (**a**–**e**) generation of cracks around particles of different sizes after bending and (**f**–**i**) larger particles that can also be passivated after bending with the addition of a planarization layer [39].

Chen et al. [21] postulated that the addition of an ALD-SiO_2_ film as a cover layer of the particle (Figure 10b) could enhance the particle passivation performance in the conventional CVD-SiO_x_N_y_/organic layer/CVD-SiO_x_N_y_ TFE structure. Due to its high step coverage (exceeding 95%), ALD-SiO_2_ is capable of forming a barrier film around the particles that is both durable and effective in preventing the permeation of water vapor. Park et al. [39] achieved particle passivation through the formation of a nanolaminated layer comprising ALD-Al_2_O_3_ and a plasma polymer n-hexane organic layer. This enabled the OLED displays to be bent at a radius of 1 mm. The authors demonstrated that the encapsulation process was reliable, as shown in Figure 11. They also found that the encapsulation remained reliable when the angle between the encapsulation films and the particle exceeded 140° in a 1mm radius folding.

#### 2.2.3. Compatibility

Although TFEs are only a very small part of the display, compatibility between TFEs and display devices also requires special attention. Indeed, the aforementioned transparency, stress matching, and reliability of TFEs are all inherently related to the impact of compatibility.

Aside from the aforementioned relations, TFEs may also influence the functionality of thin-film transistors (TFTs), which also require high stability during operation [55,56]. This is due to the fact that the most currently utilized inorganic barrier films of TFEs (i.e., SiN_x_:H) typically possess a considerable hydrogen content [34,57,58,59,60]. Moreover, in the context of an oxide TFT, the switching characteristics are susceptible to being influenced by the hydrogen that was initially present in SiN_x_:H and migrated from SiN_x_:H. In severe cases, the TFT may be directly affected by the H, resulting in direct conductance and a loss of switching ability [61,62]. Nevertheless, in some instances, a modest quantity of H doping has been observed to improve the functionality of oxide TFTs [63]. It is therefore important to reduce or control the hydrogen content of TFE materials in order to enhance the compatibility. Yin et al. [64] observed that although the change is slight, the encapsulation films with different process conditions influence the shift of the threshold voltage (Vth) by monitoring the change in the Vth of TFTs over the entire OLED display panel. The results showed that the higher the flow rate of NH_3_ gas utilized during film deposition, the more susceptible the Vth tended to deviate negatively. Additionally, there are other methods for reducing the hydrogen content of the film, including O_2_ plasma treatment [60], adding a hydrogen blocking layer [65], and modifying the reaction parameters [46].

The impact of TFEs on OLEDs is also of significant importance in determining their compatibility. Singh et al. [66] reported that ozone is a preferable reactant to water in the context of ALD-Al_2_O_3_ deposition on OLEDs. The utilization of water as a reactant resulted in the formation of severe black spots on ALD-nanolaminate Al_2_O_3_/TiO_2_ encapsulated OLEDs, while ozone exhibited a reduced propensity for spot formation. This phenomenon can be attributed to the heightened susceptibility of OLEDs to water, with damaged OLEDs displaying areas of black spots that gradually grew in size. Another report also showed that the lifetime of OLEDs may be enhanced when using ozone as a reactant in comparison to water [67]. Furthermore, it can be postulated that the hydrogen and other components in TFEs may also affect the OLED light-emitting efficiency, lifetime, and other properties of a display. This is a crucial aspect that requires verification in the engineering of large-size OLED displays.

In addition to the impact of TFEs on OLEDs/TFTs, their compatibility with neighboring films also represents a significant area of concern. Among them, the adhesion between organic and inorganic films within TFEs represents a pivotal factor influencing the lifetime of encapsulation. When the adhesion between organic and inorganic films is low, it is easy for the films to separate from each other after high temperature and high humidity or bending tests due to the inability to withstand the additional stress, thus forming a channel for water vapor intrusion [33]. Wang et al. [68] achieved enhanced adhesion between films and a more reliable OLED display by adjusting the ratio of N to Si in the PECVD-SiN_x_. The authors proposed that enhanced adhesion can be attributed to the composition of the material being more closely approached to the standard stoichiometric ratio. The addition of intermediate buffer layers and surface treatment processes represents effective methods for improving adhesion too [69]. When TFEs are used in large-size rollable OLED displays, the interlayer shear stress significantly increases, and thicker TFEs are more susceptible to shear stress, which can potentially result in shear peeling between TFEs and neighboring films, ultimately leading to display failure.

It is conceivable that TFEs may absorb the light emitted by OLEDs. When there is significant variation in film thickness or the refractive index across the entire surface of the encapsulation film, it is relatively straightforward to observe nonuniformity in display performance, which is commonly referred to as the MURA effect. In this regard, ensuring uniformity in the film thickness and composition of the encapsulated film is of critical importance to guaranteeing optimal yields in the engineering process. Furthermore, given that the sheet mask is employed to pattern the encapsulated film for the mass production of OLED displays, it is essential to address the potential for uneven film thickness near the edges of the sheet and the potential shadowing effect on the color performance of OLEDs.

### 2.3. Principles and Mechanisms of Thin-Film Encapsulation

#### 2.3.1. Barrier Principles of Thin-Film Encapsulation

In recent decades, high-specification TFE has undergone significant development, evolving numerous forms to enhance the barrier performance. Initially, the focus was on the development of single-layer barriers (mainly inorganic films), followed by the lamination of inorganic films and finally inorganic–organic laminating structures.

For single-layer barrier films, the most original barrier ability comes from the narrow interstitial void that emerges subsequent to the internal atoms or molecules being arranged in a compacted configuration [70]. When the void’s size is smaller than the size of water molecules (about 0.33 nm) [71] or oxygen molecules (about 0.32 nm), water vapor or oxygen needs a larger activation energy ($\Delta G\overset{+}{+}$) to squeeze into these voids and penetrate the film. For an ideal permeability model of gas permeating through silica glass or glass-like polymer, the Arrhenius Formula (3) can be used to express the permeability Π:

(3) $$\Pi =\Pi_{0}exp(-\Delta G\overset{+}{+}/RT)$$

where $\Delta G\overset{+}{+}$ represents the activation energy of permeation, R is the constant of molar gas, and T is the temperature. It can be seen that the permeability is related to temperature, and the higher the temperature, the greater the permeability. By changing the temperature, the activation energy of permeability can be obtained readily.

**Figure 12:**
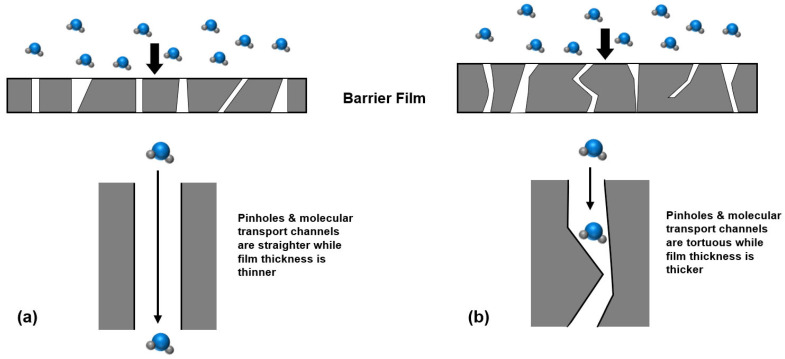
Schematic representation of (**a**) straight channels and (**b**) tortuous channels for molecular transportation.

Ideally, $\Delta G\overset{+}{+}$ relates to the interstitial void of the film; the smaller the void, the higher the activation energy required, and the smaller the permeation rate. However, in fact, most of the thin films used for the gas diffusion barrier have more or less defects (mainly referring to physical defects like pores, pinholes, etc., not chemical defects like impurities), and the type, density, size, and shape of these defects are more critical factors for $\Delta G\overset{+}{+}$, which results in a sensitive permeability to the presence of defects [70]. It is generally believed that there is a threshold thickness of barrier film [72,73,74,75]; when the thickness exceeds the threshold, the decreased rate of water vapor permeability will tend to be moderate. Erlat et al. [70] showed that the activation energy $\Delta G\overset{+}{+}$ increases when the film thickness is increased, which is distinct from the case where a strong correlation exists between the pinhole and permeation (while the defect density remains unchanged, the permeation cannot be reduced only by increasing the film thickness). This may be due to the fact that the pinholes become different in size and the channels formed by the pinholes become more tortuous as the film thickness increases, as shown in Figure 12. Accordingly, when the quantity, size, and tortuousness of the pinholes reach a statistical uniformity, the contribution of further increasing film thickness to the water vapor permeability becomes weaker.

Considering the influence of defects, Hanika et al. [76] studied the influence of defect size and spacing between defects on gas permeation modeling. On the basis of non-defect areas with the same original permeability, while we simulate there are defects in the film, small defects with high density exhibit higher permeability than large defects with low density. However, the authors also mentioned that this was only the case of gas (e.g., O_2_) permeation, and that the case of water vapor permeation is more complicated and needs further study.

For the lamination of barrier films, it has been identified by researchers previously that the lamination of inorganic barriers made from different materials or processed under different conditions exerts a profound influence on enhancing the barrier properties [72,77,78,79]. One of the commonly accepted mechanisms is that the defects between disparate films will be staggered, which will passivate the defects and lengthen the water vapor transmission paths, thus reducing the WVTR. Meanwhile, for laminated barrier films, the WVTR_total_ will be improved by the WVTR originating from multiple barrier films. If the WVTR of a single barrier film is known, the following Equation (4) [77] can be used to estimate the WVTR_total_ of the stacked film:

(4) $$\frac{1}{{WVTR}_{total}}=\frac{1}{{WVTR}_{1}}+\frac{1}{{WVTR}_{2}}+\frac{1}{{WVTR}_{3}}+\frac{1}{{WVTR}_{4}}+\ldots$$

In addition, the laminating of inorganic barrier films is not only the addition of the WVTR, but also the formation of special chemical bonds at the interface [72], the passivation of pinholes [78], and other special functionalities, which help to achieve better barrier performance.

Organic films have been an indispensable presence in today’s TFE structures, though inorganic films exhibit much higher gas diffusion barrier properties than organic ones. Considering the reason, firstly, the organic film is generally obtained from the precursor with high fluidity. The precursors exhibit a high spread capacity on the substrate during the deposition/coating process, which can play a role in flattening the substrate surface. Secondly, the organic film itself features a markedly low Young’s modulus. As a result, when the OLED display is bent, the organic layer can effectively serve as a stress buffer for its neighboring inorganic films, thereby enhancing the mechanical reliability of the OLED display. Moreover, referring to Figure 1b, similar to the principle of inorganic laminate, the formation of an inorganic/organic/inorganic encapsulation structure can also effectively increase the water vapor intrusion path and reduce the WVTR.

Nowadays, the nanolaminate consisting of ALD films, ALD/MLD films, and ALD/organic films exhibits more functionalities for better barrier performance, which will be described in detail in the subsequent sections.

#### 2.3.2. Failure Mechanisms of Thin-Film Encapsulation

The failure of TFE implies that the encapsulated OLED device is eroded by water vapor or oxygen, leading to the inability to emit light in a normal manner. Regardless of the underlying cause of TFE failure, the ultimate behavior is the generation of channels that can be transported by water vapor or oxygen. In general, the failure of TFE can be attributed to four main factors, namely: material degradation, particle contamination, mechanical brittleness or peeling, and lateral invasion [80,81].

One of the critical failure modes of TFE is material degradation. It can be reasonably assumed that, as long as the material and structural design of the TFE reaches the anticipated gas diffusion barrier capability, it is unlikely that water vapor would invade the OLED and cause failure. However, despite the excellent barrier performance of some films, they will gradually react with water vapor, resulting in the transformation of the film material [82]. These transformations commonly result in the formation of pores and channels through which water vapor can pass. SiN_x_ films prepared by PECVD are commonly used as inorganic barrier films for TFE in today’s commercial manufacturing and possess excellent barrier properties. However, depending on the process parameters of PECVD, SiN_x_ may exhibit chemical reactivity with water vapor and oxygen from the ambient environment, leading to the formation of SiO_2_ [34] and a significant deterioration in the WVTR. This results in the intrusion of water vapor, ultimately leading to failure. In general, the doping of H_2_ into the PECVD reaction gas can effectively enhance the stability of SiN_x_ films [83]. It has been explained that the introduction of H_2_ helps to increase the content of SiH_3_* and NH_2_* in the plasma, which facilitates the migration of the reaction group on the reaction surface during the film deposition process. This enables the passivation of a greater number of defects and the reduction in surface roughness as well as the specific surface area. As a result, the probability of reaction with water vapor or oxygen in the air is reduced. Additionally, the introduction of H_2_ facilitates the disruption of some of the weak chemical bonds in the reactive surfaces during deposition, thereby increasing the film densities [84]. Furthermore, atomic layer deposited alumina films (ALD-Al_2_O_3_) are of interest given their high barrier property, with a film thickness of only 50 nm, achieving a WVTR of 10^−5^ g m^−2^ day^−1^ levels [85]. However, several studies have demonstrated that alumina reacts readily with water vapor in warm and humid environments, forming alumina hydroxide [86,87,88,89]. This transformation results in the formation of a porous and swollen film, which differs from the original dense alumina and is no longer capable of effectively blocking water vapor or oxygen.

**Figure 13:**
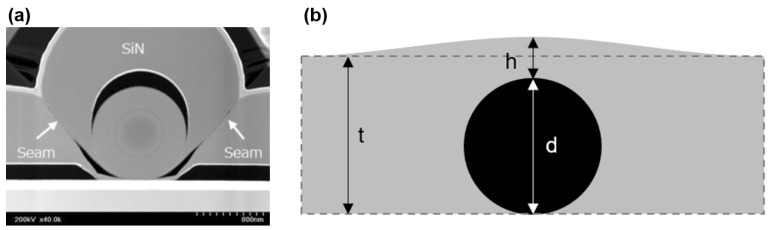
(**a**) Cross-sectional SEM image representing particle resulting in the formation of water vapor intrusion channel in the encapsulation film [16] (copyright © 2023, The Society for Information Display). (**b**) Schematic diagram of flattening performance evaluation method.

The presence of particles would inevitably affect the encapsulation performance of TFEs. However, the extent of this influence is contingent upon the size of the particles in question and the passivation ability of the TFE with regard to them. Particles are typically regarded as particulate dust originating from the surrounding environment, equipment, or sub-products generated during the fabrication process. They typically range in size from 0.1 to 500 μm. The angular nature of particles results in severe damage to inorganic barrier films, as previously discussed in Section 2.2.2 and illustrated in Figure 13a. Furthermore, the impact of particles on rollable OLED displays is likely to be more significant, given that particles have the potential to squeeze surrounding films, leading to stress concentration and subsequent cracking of the films. The rolling action would result in the bending of the entire display panel, in contrast to the bending of only minimal parts of the display panel in the case of a foldable OLED display. Consequently, the presence of particles in the display results in the formation of cracks, as illustrated in Figure 11. To mitigate the impact of particles, a flattening layer can be incorporated to passivate them, as discussed in Section 2.2.2. The performance of the flattening layer can be evaluated using the following Formula (5) [90]:

(5) $$Planarization=(1-\frac{h+d-t}{d})\times 100\%$$

Refer to Figure 13b, where d is the diameter of the particle, h denotes the distance from the uppermost point of the flattening layer to the particle, and t signifies the thickness of the flattening layer.

For flexible display products, bending, rolling, and stretching are the most common operations, depending on the different products. In order to assess the reliability of the flexible display panels, it is necessary to utilize the appropriate instruments to carry out thorough testing. In the case of the display of a foldable smartphone, for example, the process of actual use is generally simulated. The majority of the display is fastened to two rotating plates (hands) that can be opened and closed to each other, leaving the part that needs to be folded empty. The display is then folded more than 100,000 times. In this way, an evaluation of mechanical reliability is achieved. A mechanical rupture may occur if the mechanical reliability is not promising. The rupture is referred to as the irreversible plastic deformation that occurs when encapsulation films in plastic materials are subjected to stress that exceeds the film’s maximum tolerable stress, which is typically manifested as cracks. The minimum stress that results in the plastic deformation of the film is denoted as the critical strain, or the crack onset strain (COS), which is often presented as $\varepsilon_{c}$. The critical strain is related to the critical stress $\sigma_{c}$ and the Young’s modulus of the film $E_{f}$, as expressed by the following Equation (6):

(6) $$\varepsilon_{c}=\frac{\sigma_{c}}{E_{f}}$$

It can be seen that the smaller the Young’s modulus (i.e., the softer the film), the greater the strain it can withstand.

In a model of films experiencing tensile stress, and according to the Shear Lag Model, when a film of thickness h is deposited on a substrate of thickness $h_{s}$, considering the case of creating a new crack with two already existing cracks (for simplifying the calculation), the critical stress $\sigma_{c}$ can be expressed by the following Equation (7) [42]:

(7) $$\sigma_{c}=D{\left[h_{s}/\left(h/\left(h_{s}+h\right)\right)\right]}^{1/2}$$

where h is the thin-film thickness, $h_{s}$ is the substrate thickness, and D is a number of related variables, which can be expressed using Equation (8):

(8) $$D={\left(2G_{C}{\xi E}_{S}E_{f}/E\right)}^{1/2}$$

where $G_{C}$ is the critical energy release rate; the $E_{S}$, $E_{f}$, and E are the Young’s modulus of the substrate, film, and substrate–film synthesis, respectively; and *ξ* is a fitting parameter.

When utilizing TFEs, since the substrate thickness is commonly much larger than the film thickness ($h_{s}\gg h$), and the Young’s modulus is a constant of a given material, solving Equation (6), the critical strain is related to the film thickness as follows:

(9) $$\varepsilon_{c}\sim {\left(\frac{1}{h}\right)}^{1/2}$$

**Figure 14:**
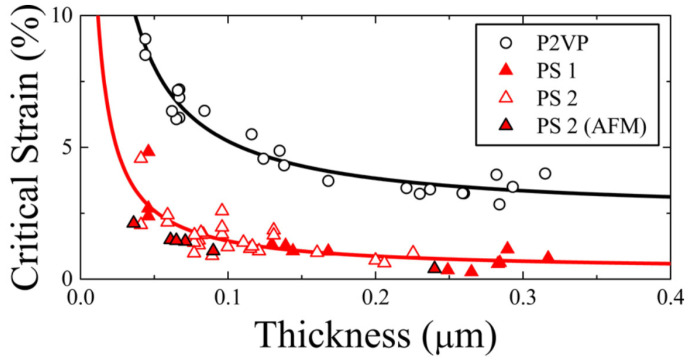
Relation between thin film thickness and critical strain [91] (copyright © 2015, American Chemical Society).

It can be seen that the smaller the thickness, the higher the critical strain, and the film is less prone to cracking. As shown in Figure 14, the measured film thickness versus critical strain (the tensile strain) for thin films is plotted, and the fitted curve basically fits Equation (9).

**Figure 15:**
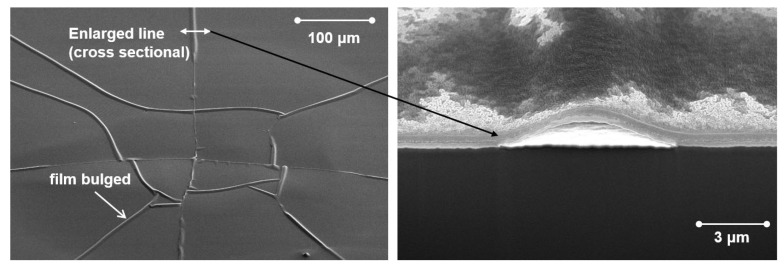
SEM image representing bulge of Al_2_O_3_ thin film at compressive strain.

The above theoretical model can well reflect the situation when cracks are caused by tensile stresses, but the situation when compressive stresses are applied will be somewhat different: the critical strain for compressive stresses is a bit higher than that for tensile stresses. This may be related to the process of crack formation by compressive stress and the way the stress is released. Compressive stress generally causes the film to bulge first (Bulking), as shown in Figure 15. However, with continued application of compressive stress, cracking may occur at the top of the bulge where the thin film is bent with tensile stress. Although there is no theoretical model to correlate the critical strain of compressive stress with film properties, the same as tensile stress, the smaller the film thickness, the greater the critical strain. And for oxide films with film thickness greater than a certain degree (e.g., ALD-Al_2_O_3_ has a thickness greater than 50 nm), the critical strains in the two stress modes are almost the same (COS ≈ 1%).

In addition to the strain caused by external forces, the intrinsic strain of the thin film itself ($\varepsilon_{0}$), the thermal mismatch strain ($\varepsilon_{th}$) between the film and the substrate or adjacent layers generated during the deposition, and stresses caused by humidity mismatches ($\varepsilon_{ch}$) also affect the critical strain, especially when they are greater than a certain level. Collectively, these are referred to as internal mismatch stresses ($\varepsilon_{m}$), which can be expressed by the following Equation (10) [92]:

(10) $$\varepsilon_{m}=\varepsilon_{0}+\varepsilon_{th}+\varepsilon_{ch}$$

The intrinsic stress has been demonstrated to be significantly correlated with the chemical bonding configuration within the film and the molecular stacking structure. The humidity mismatch stress is found to be minimal and has a negligible impact. In contrast, residual thermal stress is found to be highly correlated with the discrepancy in coefficients of thermal expansion between the film and its neighboring films. The primary mechanism responsible for residual thermal stress is that when the film is recovered to normal temperature from high-temperature deposition, since the coefficients of thermal expansion between the thin film and the neighboring films are not identical, and after cooldown, the extent of shrinkage between them is not the same, rendering the film subject to stress from neighboring films. Commonly, the thermal stress between inorganic films and organic films or substrate may result in the compressive stress of inorganic films. To consider thermal stress $\sigma_{f}$, the following Formula (11) may be referenced:

(11) $$\sigma_{f}=\left[E_{f}/\left(1-v_{f}\right)\right]\int_{T1}^{T2}\left[\alpha_{s}\left(T\right)-\alpha_{f}\left(T\right)\right]dT$$

where $\alpha_{s}\left(T\right)$ is the CTE of the substrate as a function of temperature and $E_{f}$, $v_{f}$, and $\alpha_{f}\left(T\right)$ are the Young’s modulus, Poisson’s ratio, and the CTE of the film as a function of temperature, respectively. T1 and T2 are the temperatures in normal and depositing situations, respectively. If consideration is given to applying the model to the thermal stress between inorganic/organic laminated films, and further considering the effect of thickness, the thermal stress can be calculated using the following Equation (12) [93]:

(12) $$\sigma_{f}=\frac{\left(\alpha_{0}-\alpha_{i}\right)}{d_{i}\left[\frac{\left(1-\nu_{i}\right)}{d_{i}E_{i}}+\frac{\left(1-\nu_{0}\right)}{d_{0}E_{0}}\right]}\Delta T$$

Among them, $d_{0}$ and $d_{i}$ are the thicknesses of the organic and inorganic films, respectively. $\alpha_{0}$ and $\alpha_{i}$ are the CTEs of the inorganic and organic films, respectively. ΔT=T1−T2. $E_{0}$, $E_{i}$, $\nu_{0}$ and $\nu_{i}$ are the Young’s modulus and Poisson’s ratio of the organic and inorganic films, respectively. Utilizing Equation (12), the thermal stress between thin films and the organic substrate can be calculated, between which thermal stress was most significant in a flexible OLED structure.

Currently, flexible OLED displays usually demonstrate their flexibility through the shape of bending, including static curved display, dynamic foldable display, and rollable display. Consider a bending model of the TFE layer deposited on the substrate, in the cross-sectional direction, there is always a neutral plane, as mentioned in Section 2.2.2. At this point, the strain on the TFE layer $\varepsilon_{z}$ can be expressed as Equation (13):

(13) $$\varepsilon_{z}=\left(\frac{Z-Z_{n}}{R}\right)$$

where $Z_{n}$ is the position of the neutral layer, Z is the position of the TFE layer, and R is the bending radius. Assuming that the Young’s modulus of the TFE layer is the same as that of the substrate, the neutral plane will be at the geometric center of the model, at which point the strain on the upper surface of the model (i.e., the uppermost layer of TFE) can be expressed as Equation (14):

(14) $$\varepsilon_{top}=\frac{d_{1}+d_{2}}{2R}$$

Among them, *d*_1_ and *d*_2_ are the film and substrate thickness, respectively. If Young’s modulus is also taken into account, Equation (14) needs to be adjusted to the following Equation (15):

(15) $$\varepsilon_{top}=\left(\frac{d_{1}+d_{2}}{2R}\right)\frac{\left(\chi \eta^{2}+2\eta +1\right)}{\left(1+\chi \eta \right)\left(1+\eta \right)}$$

Among them, $\chi =E_{1}/E_{2}$, $\eta =d_{1}/d_{2}$. *E*_1_, and *E*_2_ are the Young’s modulus of the film and substrate, respectively. Utilizing the above equation, the magnitude of strain applied to the uppermost layer in the bending model can be estimated.

If we consider a more complex bending model that comprises a multilayer and multi-material thin-film laminate, the position of the neutral plane $Z_{n}$ is associated with the Young’s modulus and thickness of each film, which can be expressed by the following Equation (16):

(16) $$Z_{n}=\frac{\sum_{i=1}^{n}E_{i}d_{i}^{2}+2\sum_{i=1}^{n}\left(E_{i}d_{i}\sum_{j=1}^{i-1}d_{j}\right)}{2\sum_{i=1}^{n}E_{i}d_{i}}$$

where i is the index of the etch film. Utilizing this equation, the position of the neutral plane can be adjusted by the Young’s modulus and thickness of each layer, so as to set the position at the most mechanically weak film. Thus, the mechanical reliability of the whole system can be ensured.

**Figure 16:**
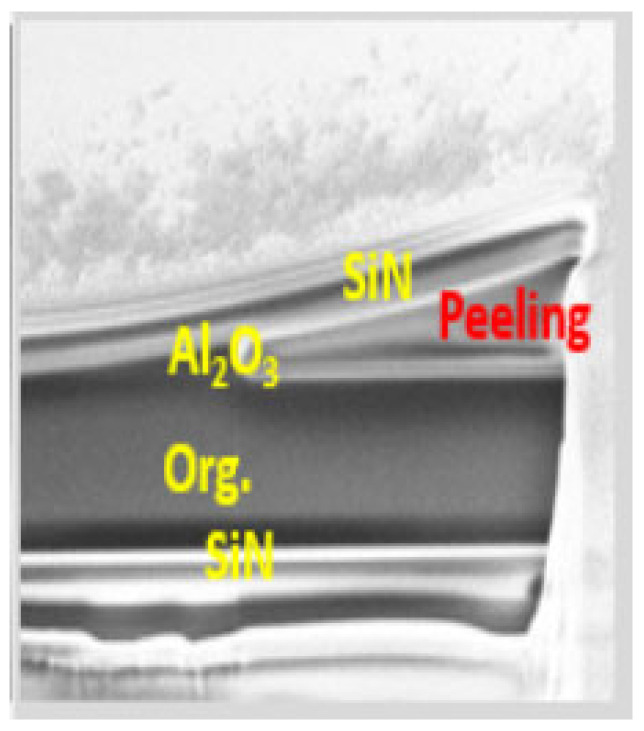
Cross-sectional SEM image of peeling due to low adhesion (copyright © 2019, The Society for Information Display).

Aside from the mechanical rupture, mechanical peeling is also a common mechanical failure mode of OLED displays. One of the fundamental reasons for this is that OLED displays are stacked with a variety of organic/inorganic films that are deposited at low temperatures. The adhesion of films in such devices is generally much lower than those devices comprising high-temperature-deposited and all-inorganic films. For OLED rollable display products, the shear stresses between the etch layer can be very large at the edge of the display (like a rolled book). Therefore, improving the adhesion between organic and inorganic films in the TFE structure and improving the adhesion between TFEs and the organic layer of OLEDs are very important tasks in the development of extremely flexible OLED displays. Usually, the adhesion force can be improved by material matching between adjacent layers, surface treatment, reduction in residual stress, and the addition of a transition layer. Tianfu Guo [33] et al. attempted to introduce an ALD layer into a CVD/organic/CVD TFE structure. ALD-Al_2_O_3_ exhibited high stress and poor adhesion with the organic film, which resulted in a peeling-off phenomenon after the reliability test (as shown in Figure 16). By adding a SiO_x_ layer between ALD-Al_2_O_3_ and the organic film, the adhesion between them can be effectively improved, thus obtaining higher reliability.

**Figure 17:**
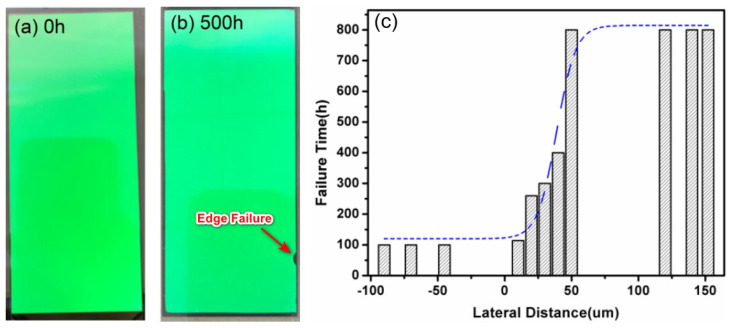
(**a**,**b**) Schematic of edge failure of OLED display after high-temperature and high-humidity storage test. (**c**) Plot of lateral distance between the edge of the encapsulation and the OLED active area versus the time to failure (copyright © 2019, The Society for Information Display).

Lateral intrusion is a kind of failure mode that often needs to be considered in the engineering period. In the OLED display, the film encapsulation achieves complete protection by wrapping the OLEDs. Since the encapsulation film is deposited on the substrate in the form of a layer, at the edge of the OLED display area, it is necessary to laminate the TFEs with a layer that possesses the same barrier performance on the substrate side below the OLEDs to form a complete encapsulation structure encircling the OLEDs. However, because the interface between the multilayer films is exposed to the normal environment at the edge, water vapor can easily invade from the interface. When water vapor permeates through the interface between the barrier layer and the OLED, the failure area (the dark area) first appears along the edge and then grows laterally, which is the edge failure in contrast to the radial growth due to the pinhole defects, as shown in Figure 17.

**Figure 18:**
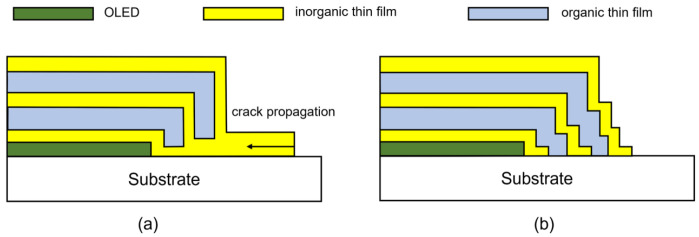
(**a**) Traditional TFE edge structure. (**b**) Optimized TFE edge structure for inhibition of crack propagation utilizing organic-layer block.

In order to reduce edge failure, it is essential to ensure that there is a sufficiently long distance between the lateral edge of OLEDs (the active area) and the TFEs. This is illustrated in Figure 17c, which demonstrates the relationship between the failure time and the distance of the encapsulation display edge [68]. On the other hand, designing an appropriate TFE edge structure so as to reduce the exposure area of the interface and strengthen the material suitability between TFE and the barrier film at the substrate side, thus increasing the adhesion of the interface, can also improve the encapsulation reliability. Seung Woo Lee et al. [94] demonstrate an effective method for passivating edge cracks by modifying the stacking structure of inorganic/organic films at the edges, as illustrated in Figure 18. This approach effectively inhibits the lateral expansion of edge cracks, which may be caused by the cutting process, thereby enhancing the reliability of encapsulation.

## 3. Novel Thin-Film Encapsulation Technology

**Figure 19:**
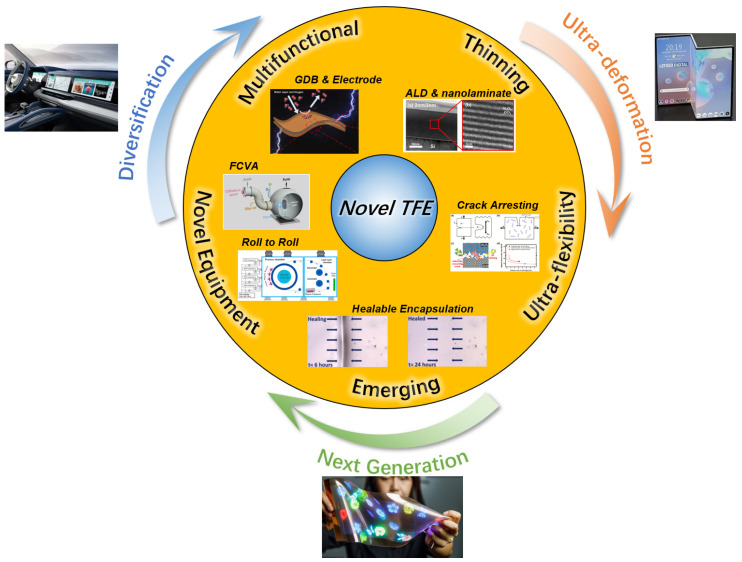
An overview of novel TFE technologies in terms of thinning, ultra-flexibility, novel equipment, multifunctional, and emerging technologies. Each technology is driven by the corresponding product type.

As the commercialization of OLED display products becomes increasingly prevalent, and more importantly, as the rivaling technologies (such as flexible micro-LED) keep emerging, the development trends of OLED displays can be observed to have full-size coverage, have diversified forms, be thin/light, and have extreme flexibility. Through achieving the above-mentioned innovation, OLED displays shall keep their own advantages and, meanwhile, develop further excellent products. Although TFE technology represents only a minor component of overall OLED display technology, given the ongoing evolution and refinement of OLED display products, the current TFE technology still exhibits considerable potential for enhancement. Consequently, the progression of novel TFE technology in engineering applications is also accelerated. As illustrated in Figure 19, it can be postulated that the thinning and ultra-flexible technology of TFE driven by ultra-deformation products will constitute a pivotal development trajectory for novel TFE. The multifunctionalities and novel equipment technology driven by the demand for diversification and full-size covering of products such as automotive, laptop, monitor, etc., will represent a new value growth point for TFE. Furthermore, the encapsulation performance required for next-generation display products (stretch display, fabric display, etc.) is also driving more R&D outcomes that can break through traditional concepts to support the development of emerging encapsulation technologies.

### 3.1. Thinning Technology

The current flexible encapsulation solutions used in commercial manufacturing are typically inorganic/organic/inorganic stacked-layer encapsulation, in which the inorganic layer mainly refers to inorganic films of silicon nitride (SiN_x_) and silicon oxynitride (SiO_x_N_y_) prepared by plasma-enhanced chemical vapor deposition (PECVD) technology, which have a high water vapor/oxygen barrier property and the thickness is generally greater than 1000 nm [95]. The organic layer mainly refers to transparent polymer films prepared by inkjet printing (IJP) technology, which have excellent defect coverage and stress buffer performance, and the thickness is generally set to 8~15 μm. From the perspective of the overall structure of the TFE, its thickness generally requires 9~17 μm.

According to the theory of neutral planes, as mentioned in Section 2.2.2., in order to ensure the normal functions of the flexible display, the neutral plane is generally intentionally set in the position of fragile anode or the thin-film transistors (TFTs). Therefore, the TFE layer might be away from the neutral plane; the thicker the TFE is, the stronger the stresses it is subjected to, and the more likely to generate cracks and failure. In addition, due to the presence of intrinsic stress, when the film is thicker, the greater the film stress G will be (according to the formula G = σ·h) [23], and the greater the strain on the adjacent film and its own will be. Therefore, in order to achieve a smaller bending radius, the thinning of TFE is a very important direction of research and development.

Currently, the thickness of inorganic barrier layers generally needs to be more than 1000 nm in order to ensure the reliability of OLEDs for a long period of time; however, due to the fact that the inorganic barrier layer generally has a large Young’s modulus, it is easy to rupture and crack during the bending process. Especially, while referring to the outer inorganic layer of TFE, its position is further away from the neutral plane and will be more likely to crack due to the more powerful mechanical strain. This is why, for most flexible OLED display products, it cannot satisfy the demand of a bending radius of <1 mm rather than >2.5 mm.

**Figure 20:**
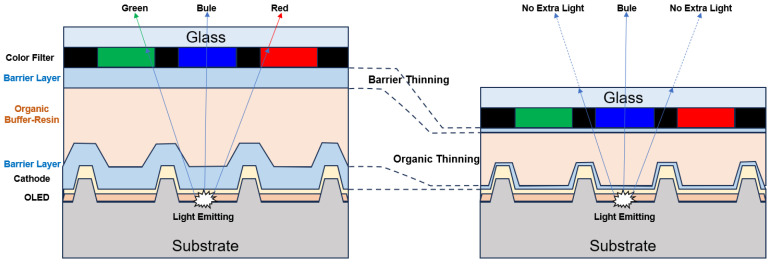
Schematic representation of the demand for TFE thinning in high-resolution OLED displays.

On the other hand, with the development of head-mounted and head-up display products such as VR/AR/MR/HUD, the requirement for display resolution is getting higher and higher. In this context, as illustrated in Figure 20, Micro-OLEDs containing a color filter require that the spacing between the OLED pixels and the CF should not be too large, or else crosstalk is likely to occur, thus affecting the color purity of the display [16,96]. This also requires that the TFE layer should be thinned (~1 μm).

In order to achieve a smaller bending radius and superior display performance, the development of TFE has been undergoing a prolonged process of thinning, and Table 1 illustrates a great deal of research findings pertaining to the thinning of barrier film in terms of its various properties while undergoing engineering verification.

#### 3.1.1. Monolayer of ALD Barrier Film

**Figure 21:**
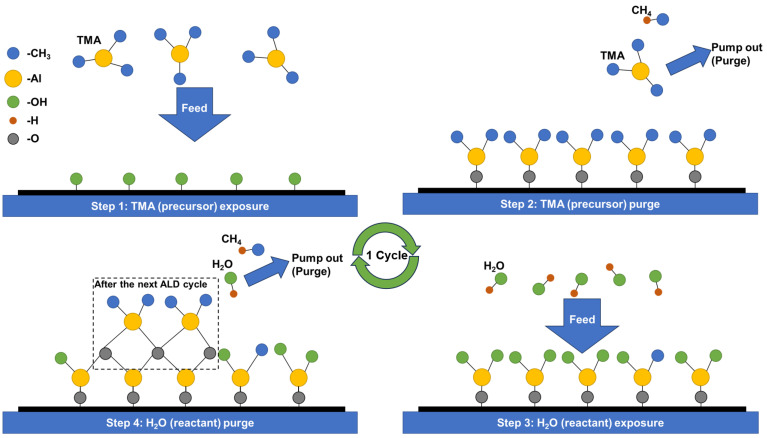
Schematic diagram of the growth process of commonly used ALD (Al_2_O_3_).

ALD is a widely studied thin-film deposition technique and is considered to be the most likely replacement for PECVD deposition in TFE. ALD is a self-limiting chemical vapor deposition technique, as shown in Figure 21. The fundamental process involves the following: precursor feed and adsorption, precursor purge, reactant feed and reaction, and reactant purge. By cycling through the fundamental processes, materials are deposited on the substrate at an atomic or molecular level and gradually increase in thickness, which is where the name atomic layer deposition comes from.

Contributing to its self-limiting reaction properties, the ALD layer exhibits the advantages of dense films, fewer pinholes, high uniformity, good conformality, finely controllable thickness, and low-temperature deposition. As a result, atomic layer deposited films can achieve an excellent water vapor/oxygen barrier ability at a thin thickness. In addition, due to the wide variety of precursors (generally metal compounds) required for its reaction process, theoretically, any material can be utilized in ALD as long as the precursor can be adsorbed onto the deposition target surface after transportation. It is only in practice that the adsorption capacity between the substrate and the precursor affects the ability of some materials to achieve good densification.

Al_2_O_3_ is one of the most widely researched and applied materials for ALD. As an encapsulation barrier film, Al_2_O_3_ films with a thickness of 30~100 nm are comparable to PECVD-SiN_x_ films with a thickness of 500~1000 nm. The precursor for ALD-Al_2_O_3_ films is generally trimethylaluminum (TMA), which is relatively stable and readily available, and can be adsorbed onto a variety of substrates of different materials. The reactants are generally H_2_O, O_3_, and plasma O_2_ (O_2_ plasma), of which the O_3_ and plasma O_2_ processes can obtain films with better barrier properties [85,97], which is more related to the lower content of -OH bonds in the reaction products. Plasma O_2_, as a reactant, can reduce the deposition time significantly, because H_2_O and O_3_ tend to remain on the chamber sidewalls after being passed into the chamber and are difficult to be purged, and thus the purge process requires a relatively long time (10–120 s/cycle). In addition, the simultaneous use of N_2_ and O_2_ as reactants in the plasma-enhanced atomic layer deposition (PEALD) process resulted in the formation of AlO_x_:N thin films with stronger corrosion resistance [112].

In addition to Al_2_O_3_, ALD ZrO_2_, TiO_2_, and MgO have also been reported as barrier layers; however, the WVTR of all these films are relatively high, mainly due to the fact that these films are prone to crystallize during the growth process to form polycrystalline films, and there are a large number of grain boundaries in the polycrystalline films, which tend to form channels for the water vapor or oxygen [113].

According to the principle of the self-limiting reaction, the key to enhancing the density of ALD films lies in enhancing the adsorption density of precursors on the substrate and the degree of reaction between precursors and reactants. Increasing the substrate temperature is the most direct and effective way to enhance the density of ALD films, and it is generally believed that the increase in temperature can effectively reduce the hydrogen content within the film and thus enhance the density. ALD films generally exhibit a large intrinsic stress (about 300 MPa), and the increase in temperature can also reduce the intrinsic stress to a large extent [114], thus enhancing the mechanical reliability. Dung-Yue Su et al. [74] obtained a substrate densely packed with -COOH bonds on the surface by the KOH treatment, which improves the density and water vapor barrier of ALD films. Hyun-Gi Kim et al. [115] compared the film compositions before and after plasma treatment with Ar and O_2_ and found that the WVTR of the plasma-treated Al_2_O_3_ film was significantly reduced due to the fact that the plasma treatment can effectively reduce the -OH component in the film and enhance the bonding strength of the Al-O bonding. This pretreatment is effective for both PET, PEN, and PES, which are commonly used flexible substrates, where the effect of O_2_ plasma treatment is more obvious. Moreover, varying the time, sequence, and frequency of precursor feed [114] can also adjust the amount of precursor adsorption [74] and could also fine-tune the intrinsic stress of the film.

**Figure 22:**
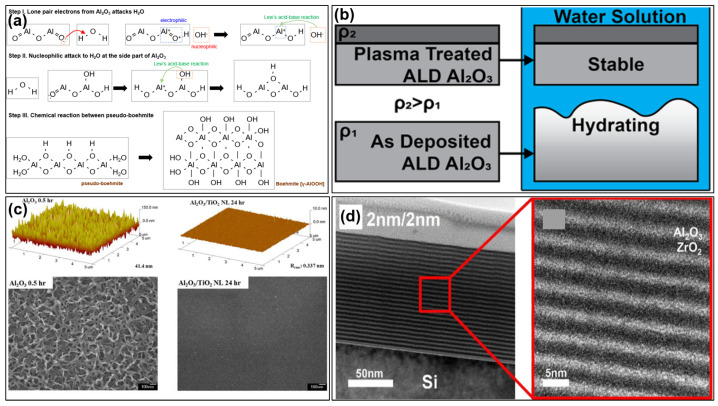
Schematic diagram of (**a**) ALD-AlO_x_ hydrolysis mechanism; (**b**) APP treatment for ALD Al_2_O_3_ (copyright © 2021, American Chemical Society); (**c**) AFM and SEM images for demonstration of ALD-monolayer and ALD-nanolaminate after RA test (copyright © 2014, American Chemical Society); (**d**) cross-sectional TEM images for illustration of ALD-Al_2_O_3_/ALD-ZrO_2_ nanolaminate [105] (copyright © 2013 Elsevier B.V. All rights reserved).

Despite the excellent water vapor barrier properties exhibited by ALD-Al_2_O_3_, the single-layer Al_2_O_3_ film is susceptible to hydrolysis when subjected to harsh environments, in accordance with Formulas (17) and (18). The hydrolysis of the Al_2_O_3_ film results in a number of observable changes, including fluffiness, an increase in thickness, and a significant reduction in barrier properties. This presents a crucial challenge for the effective functioning of the barrier layer. Figure 22a illustrates the hydrolysis process of Al_2_O_3_ [116]. Initially, the unshared electron pairs in Al_2_O_3_ are subjected to an attack by H_2_O, resulting in a Lewis acid–base reaction. The reaction results in the production of pseudo-boehmite and hydroxyl ions (OH^−^), which subsequently engage in a chain reaction by attacking the Al=O bond on the side of the molecule. The positive charge on the oxygen atoms and the negative charge on the aluminum atoms of the pseudo-boehmite molecule result in a phase transition to boehmite (Al(OH)_x_) upon reaction. Although ALD-Al_2_O_3_ is amorphous, it crystallizes during the phase transition to Al(OH)_x_. During this transition, the molar volume of Al expands due to crystallization, resulting in an increase in the total thickness of the structure. The WVTR is significantly elevated as a consequence of the grain boundaries present in the polycrystalline structure, which act as infiltration pathways for water vapor or oxygen:

Al_2_O_3_(s) + 6H^+^(aq) + 3H_2_O(l) → 2[Al(H_2_O)_3_]^3+^ (17)

Al_2_O_3_(s) + 2OH^−^(aq) + 3H_2_O(l) → 2[Al(OH)_4_]^−^ (18)

One of the mitigation strategies for the hydrolysis problem is to add a capping layer on the Al_2_O_3_ film to prevent the direct contact of water vapor with the Al_2_O_3_ film [92]. Bulusu et al. [117] investigated the ability of oxides of the metals Ni and Ti to mitigate the hydrolysis of Al_2_O_3_, which were obtained by natural oxidation, the plasma oxidation of O_2_, and PEALD. The results showed that TiO_2_, regardless of the method used to prepare it, could effectively inhibit the hydrolysis of Al_2_O_3_. Eun Gyo Jeong et al. [116] compared the effect of organic coverings with different SiO_2_ contents on mitigating the hydrolysis of Al_2_O_3_. Among them, the organic film with the highest SiO_2_ content can inhibit Al_2_O_3_ passivation to a greater extent. The role of SiO_2_ in the encapsulation structure of this film is that when the film is immersed in water, SiO_2_, Al_2_O_3_, and their hydroxides at the interface react to form a high-density and high-stability Si-O-Al bond, which effectively slows down the hydrolysis of Al_2_O_3_.

An Atmospheric Pressure Plasma (APP) treatment is a simple and easy-to-implement surface treatment process. Willis et al. [118] utilized this process for the post-treatment of ALD-Al_2_O_3_, which can effectively alleviate the hydrolysis of Al_2_O_3_ (e.g., Figure 22b). By studying the changes in film thickness and AlOOH/Al(OH)_3_ composition, the authors found that the main mechanism of the APP treatment was to increase the density of the film rather than changes in the chemical composition.

#### 3.1.2. Nanolaminate Barrier Film

Nanolaminate films are an advantageous demonstration of ALD’s ability to control film thickness at the atomic level, which not only effectively mitigates hydrolysis and enhances the critical strain of the barrier film but also enables precise control of the film’s doping composition.

Kim et al. [104] compared the roughness and surface morphology of the monolayer Al_2_O_3_ and Al_2_O_3_/TiO_2_ nanolaminate after immersion in water at 90 °C. As shown in Figure 22c, the monolayer Al_2_O_3_ formed a large number of voids on its surface, and the roughness increased dramatically after 0.5 h of water immersion, whereas the Al_2_O_3_/TiO_2_ nanolaminate was able to maintain a nice surface condition after 24 h of immersion. The authors concluded that the nanolaminate layers were able to mitigate the hydrolysis effect mainly due to the following reasons: (a) the Al_2_O_3_/TiO_2_ nanolaminate deposition formed a much denser film, and (b) the Al-O-Ti chemical bonds formed within the film were able to act as a stronger stabilizing agent than Al_2_O_3_.

To illustrate, consider the fabrication of the Al_2_O_3_/TiO_2_ nanolaminate, wherein the basic cycle of ALD-TiO_2_ is incorporated into the basic cycle of ALD-Al_2_O_3_. The combination of multiple cycles of Al_2_O_3_/TiO_2_, formed according to a specific ratio, will result in the formation of a super cycle, and the coating of the super cycle is repeated until the target film thickness is reached, thereby completing the growth of the nanolaminate.

By modifying the ratio of the constituent materials in the nanolaminate, the composition of the stack can be precisely regulated, thereby conferring versatility upon the regulation of the properties of the nanolaminate and increasing the process window. Moreover, this growth method has been observed to effectively suppress the crystallization tendency of the initial TiO_2_ film, which is prone to crystallization with increasing thickness. The incorporation of an Al_2_O_3_ film has been identified as a key factor in this inhibition. Similarly, the mechanisms of other nanolaminates, including ZrO_2_/Al_2_O_3_ (shown in Figure 22d), AlO_x_/SiO_x_ [72,119], ZnO/HfO [110], and Al_2_O_3_/ZnO [120], have been investigated, and the resulting barrier properties are presented in Table 1.

Since the WVTR values in Table 1 are measured in environments of different temperature and humidity, it is not possible to visually compare the water vapor/oxygen barrier properties of nanolaminate deposited with different materials, but it can be seen that with the increase in the cycles in the nanolaminate, a variety of materials can achieve a low WVTR. Several studies have suggested that increasing the number of laminates also increases the film interfaces, thus lengthening the water vapor intrusion paths. Furthermore, engineering applications are primarily concerned with the barrier performance of films in high-temperature and high-humidity environments. Only a limited number of studies have reported the results of the RA test, but it can be seen that Al_2_O_3_/ZrO_2_ [107] and Al_2_O_3_/TiO_2_ [28] nanolaminates can be achieved without failure for 300 and 2009 h, respectively, in environments of 85 °C/85% RH and 60 °C/90% RH. By employing the acceleration factor, 2009 h at 60 °C/90% RH can be converted to approximately 500 h at 85 °C/85% RH. This provides a preliminary indication of the relative performance of the materials under comparison. And, the Al_2_O_3_/TiO_2_ nanolaminate exhibits the most superior barrier property and reliability. However, the visible light transmittance of these films is relatively low (approximately 80%), primarily due to the low transmittance of TiO_2_ nanoparticles. In contrast, the transmittance of Al_2_O_3_/ZrO_2_ can reach up to 90%.

#### 3.1.3. Other Ultra-Thin Barrier Films

In addition to ALD deposition, several other valuable ultra-thin barrier film deposition techniques have also been reported.

By modifying the PECVD parameters, Park et al. [83] prepared silicon oxynitride (SiON) films with a reduced film thickness and a low WVTR. The results of the testing demonstrated that the WVTR of 80 nm SiON films was less than the detection limit (5 × 10^−5^ g m^−2^ day^−1^) under conditions of a high H_2_ flow ratio. The transmittance and influence on OLED devices are comparable to those of the commercially utilized PECVD thick film, and it can therefore be used as a water vapor barrier film in TFE thinning applications. However, the calculated deposition rate is 8~10 times lower than that of the conventional PECVD process, at approximately 43 nm/min, which may limit its possibility of being utilized.

Choi et al. [121] prepared SiO_2_/Al_2_O_3_ laminated barrier films by using dual-gun magnetron sputtering equipment. Utilizing transmission electron microscopy (TEM), the authors observed that the introduction of two distinct films resulted in a notable enhancement in the barrier properties against water vapor intrusion. Following the addition of a protective resin layer, the structure demonstrated the capacity to enable R10 mm and 1000 times folding, while exhibiting a minimal degree of WVTR deterioration, increasing from 3.79 × 10^−5^ to 1.64 × 10^−3^ g m^−2^ day^−1^. It is noteworthy that SiO_2_ appears to exhibit a strong affinity for Al_2_O_3_ films. Buchwalder et al. [89] conducted a comparative analysis of the structures formed when an ALD-Al_2_O_3_ was deposited between SiO_2_ or SiN_x_ films. The results demonstrated that the barrier property of ALD-Al_2_O_3_ coated with SiN_x_ was 7 × 10^−3^ g m^−2^ day^−1^. However, in the case of SiO_2_, the barrier property can reach 2 × 10^−4^ g m^−2^ day^−1^. Furthermore, the combination of ALD-SiO_2_ and ALD-Al_2_O_3_ can also result in a low WVTR [72]. Considering the mechanism, it was postulated that this was related to the formation of a more stable Si-O-Al bond between the two films.

Hong-Kyu Seo et al. [111] employed two-dimensional nanomaterial graphene as a TFE. Theoretically, graphene’s atomic spacing is smaller than that of water vapor and oxygen molecules, rendering it an optimal barrier. However, graphene films exhibit a high defect density due to the low-temperature CVD process, resulting in a poor barrier performance. As an elementary solution, the authors utilized six layers of graphene stacked to effectively enhance its barrier properties.

ALD may be regarded as the most extensively researched technique in the context of TFE thinning. From the perspective of the specifications, a monolayer of Al_2_O_3_ is already capable of meeting the requirements for the WVTR. Furthermore, the harsh environment and bending reliability can be enhanced by incorporating the nanolaminate technique. However, several issues remain to be addressed before ALD can be introduced into practical applications. One of the most crucial challenges is the low deposition rate of ALD. The current deposition rate of PECVD is 200~300 nm/min, whereas the existing reported ALD barrier process is basically less than 0.5 nm/min. It is theoretically possible to increase the deposition rate significantly by using O_2_ plasma as a reactant. However, the progress of research on encapsulated films deposited by PEALD is still relatively limited. Furthermore, among the various materials used in ALD barrier films, Al_2_O_3_ has demonstrated an exceptional barrier performance and is a widely utilized material. Nevertheless, Al_2_O_3_ is not suitable for use as a self-cleaning process, which is one of the advantages of PECVD-prepared silicide, which can be etched by fluorine plasma to enable a self-cleaning process for the chamber. Consequently, the ALD deposition chamber must be maintained with great regularity, as must the mask used for TFE patterning. This results in a considerable increase in production costs. Some other novel ultra-thin barrier techniques have been developed, which address the shortcomings of ALD, but they are still in the laboratory research stage, pending full engineering validation of these techniques.

### 3.2. Ultra-Flexibility Technology

Theoretically, in addition to the thinning of the barrier layer, the thinning of the organic buffer layer can also significantly reduce the strain on TFE layers during bending operations. However, the organic buffer layer must also fulfill the function of covering the particles, which necessitates a compromise between the passivation capability of particles and mechanical reliability. The current commercial manufacturing process employs inkjet printing equipment with piezoelectric printheads to achieve the coating and patterning of the organic buffer layer. It is challenging for this type of inkjet equipment to form a layer with a thickness of less than 2 μm without the formation of the MURA effect in the organic buffer layer. Moreover, in the context of some high-value OLED display products, it is often the case that a capacitive touch panel will continue to form subsequent to the production of TFEs. If the organic buffer layer is insufficiently thick, the effect of the parasitic capacitance may become significant, thereby interfering with the feedback of the touch signal. It is therefore necessary to consider the potential consequences of thinning the organic buffer layer against a number of factors.

Table 2 provides an overview of the extensive research conducted with the objective of achieving ultra-flexibility in TFEs. In order to investigate the potential applications of these technologies in engineering, this paper presents a comprehensive analysis of the properties that are essential for the TFE to function. As demonstrated, in terms of ultra-flexibility, the primary research directions for the development of ultra-deformable display products are the improvement of the mechanical reliability of the inorganic barrier layer and the skillful utilization of the characteristics of different organic materials to ensure the barrier and flexibility of TFEs.

#### 3.2.1. Crack Passivation Technology

**Figure 23:**
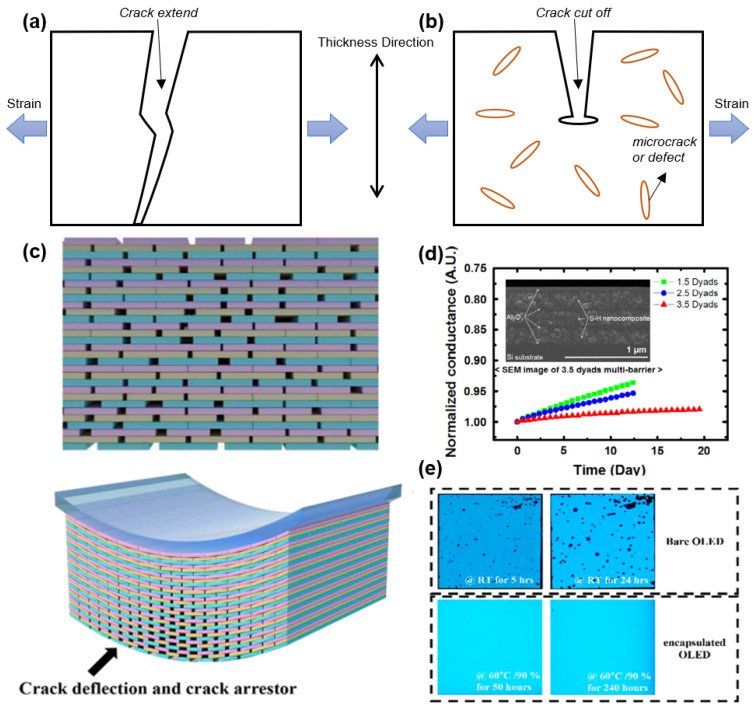
(**a**,**b**) Schematic of the passivation mechanism of natural microcracks against mechanically induced cracks; (**c**) microcracks in Al_2_O_3_/ZnO/MgO nanolaminates and their mechanism of function: microcracks generated by the corrosion of ZnO during the deposition of Al_2_O_3_ develop centers to trap and buffer the cracks (copyright © 2017, American Chemical Society); (**d**,**e**) encapsulation performance of Al_2_O_3_ with S-H organic layer and its reliability performance under 60 °C/90% R.H. environment for OLED displays (copyright © 2013, Elsevier B.V. All rights reserved).

In addition to their water vapor barrier properties, some nanolaminates can also provide special effects in terms of their mechanical properties. As illustrated in Figure 23a–c, Jeong et al. [120] demonstrated that microcracks present in Al_2_O_3_/ZnO can effectively serve as crack passivation, as evidenced by a comparison of the bending reliability of single-layer Al_2_O_3_ with that of the nanolaminate Al_2_O_3_/ZnO. Consequently, even when the film is subjected to considerable stress during the bending process, the actual stress generated can be reduced by the formation of cracks. Furthermore, these cracks can be cut off inside the nanolaminate, thus preventing the propagation of cracks through the entire film in the thickness direction, unlike in the case of single-layer Al_2_O_3_. Jeong et al. [132] from the same research group investigated the encapsulation properties of an ALD nanolaminate comprising three materials: Al_2_O_3_/ZnO/MgO (ZAM). This nanolaminate can withstand very high stresses by intentionally forming voids and defects inside the film. These defects can be used to passivate cracks during the bending process. Furthermore, the voids and defects are distributed randomly between each layer with distinct material, forming a lengthened water vapor/oxygen passage channel, which can reach a WVTR of 2.06 × l0^−6^ g m^−2^ day^−1^.

#### 3.2.2. Organic/Inorganic Nanolaminate

The organic film displays excellent flexibility, and its precursor exhibits markedly high fluidity during deposition. This can facilitate the formation of a flat deposition surface for the inorganic barrier layer, an interface that delays the water oxygen invasion rate, pinhole passivation, hydrophobicity, and other functions. The organic layer can achieve a range of functions through the modification of its functional groups and the polymerization of diverse monomers, while various organic compounds can attain exceptional flexibility and barrier properties through different mechanisms. Thus, laminating organic and inorganic films has shown great advantages, especially in flexible encapsulation.

S-H nanocomposite is a composite organic material based on Cycloaliphatic Epoxy Resin [129]. The most important feature of S-H nanocomposites is that they contain silicon oxide nanoparticles; about 19nm silicon oxide particles can be evenly dispersed in the organic matrix. Due to the existence of these silica particles, the organic film can increase the water vapor invasion path and effectively improve the barrier performance. As illustrated in Figure 23d,e, the S-H nanocomposite and ALD laminated structure retains its high barrier capacity at 1.25% (R = 6 mm) tensile strain, and its encapsulation reliability is demonstrated through storage at 60 °C/90% RH for 240 h without failure [133].

**Figure 24:**
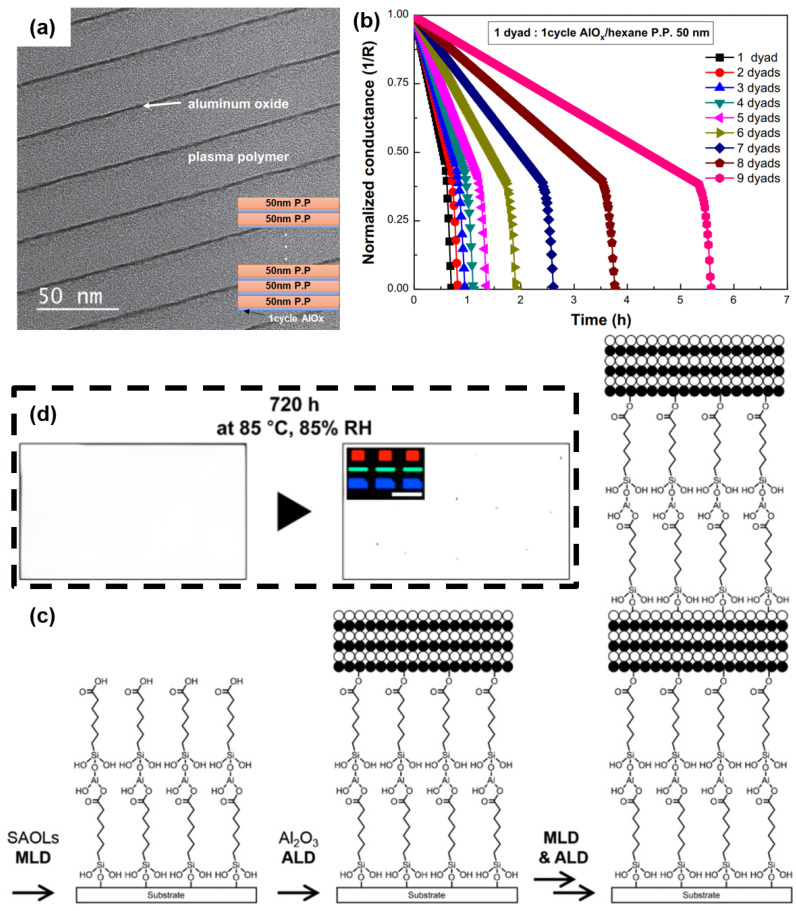
(**a**) Schematic diagram and TEM image of plasma polymer and ALD-Al_2_O_3_ nanolaminate. (**b**) Plot of the relationship between the number of nanolaminates and the barrier property [138] (copyright © 2017, American Chemical Society). (**c**) Schematic diagram of the formation of organic/inorganic stacks of MLD-SALOs and ALD-Al_2_O_3_, with (**d**) plot of the reliability test results of OLEDs encapsulated with MLD/ALD stacked structure (copyright © 2017, American Chemical Society).

A plasma polymer is a type of organic material that can be derived from the PECVD process. The formation of a thin film of plasma polymer is contingent on the process of plasma decomposition and plasma polymerization, in which vaporizable organic monomers are involved. The properties of this film, including its thickness and composition, can be tailored to meet specific requirements. It can be integrated with ALD deposition equipment within the same chamber. This feature enables the fabrication of nanolaminates comprising a significant number of layers with both ALD inorganic and plasma polymer organic films, thereby enhancing the flexibility and barrier properties of the nanolaminate. As shown in Figure 24a,b, Seung-Woo Seo et al. prepared a nanolaminate comprising ALD Al_2_O_3_ and plasma polymer hexane [127,128,138]. The research demonstrated that, under equivalent thickness conditions, an increase in the number of cycles of ALD and plasma polymer films resulted in a decrease in the sustainable bending radius. The inorganic/organic laminate of 200 pairs (with each pair consisting of one cycle of ALD and 20 nm plasma polymer films) exhibited a WVTR deterioration of less than 20% from the original after R = 5 mm bending. However, the laminate began to deteriorate rapidly after 44 h of storage in the environment at 85 °C/85% R.H.

MLD is a derivative technique of ALD. The principal distinction between MLD and ALD is that the feeding material in MLD is an organic reactant. Following a number of MLD processes, metal–organic thin films comprising carbon chain linkages can be produced. As illustrated in Figure 24c,d, Kwan Hyuck Yoon et al. [80] employed 7-octenyltrichlorosilane (7-OTS) to generate a self-assembled MLD-SALOs thin film. The encapsulated structure, formed through stacking with ALD films, demonstrated the capacity to maintain a WVTR at the level of 10^−6^ even after R10 mm bending. The authors applied the encapsulation structure to OLED displays, and the reliability test in an environment of 85 °C/85% RH showed no obvious signs of failure after 720 h, which is an impressive result in terms of reliability and flexibility among the novel TFEs that have been reported so far. If the drawback of the slow deposition rate of MLD and ALD can be effectively addressed, this encapsulation method will have significant engineering application potential.

The composite organic pV3D3 was prepared by the initiated chemical vapor deposition (iCVD) process. This film has high purity, low contaminants, as well as excellent surface planarization and pinhole passivation. Bong Jun Kim et al. [124] prepared a stack of ALD-Al_2_O_3_ with pV3D3 in the same chamber, which was able to withstand 720 h of high-temperature and high-humidity environment operational reliability tests, with only very slight degradation after R30 mm bending 1000 times. The total thickness of the stack is relatively thin (750 nm), and it is hypothesized that it can be further developed for enhanced flexibility.

**Figure 25:**
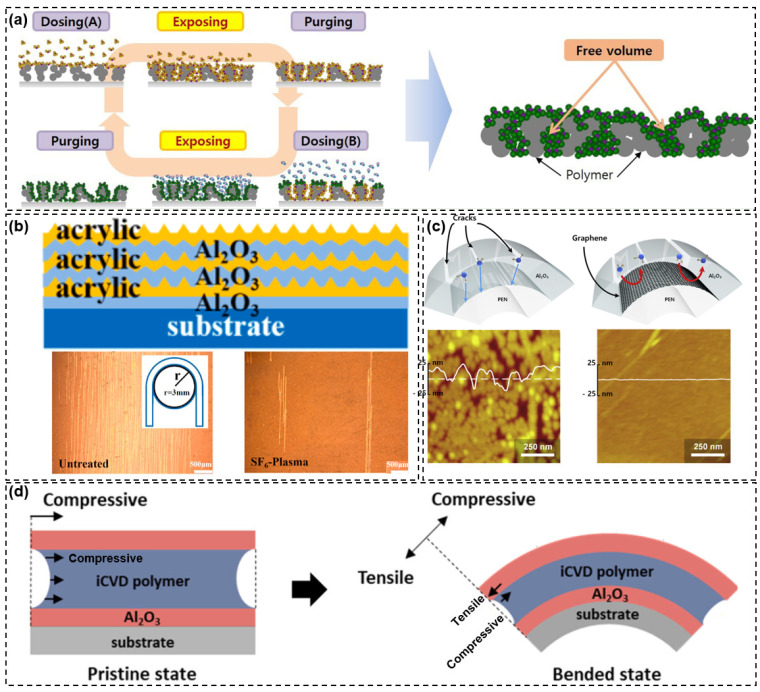
(**a**) Schematic diagram of ALI principle; (**b**) schematic diagram of PMMA fluorination treatment to form a structure contributing to bending reliability (copyright © 2021, Elsevier B.V. All rights reserved); (**c**) schematic diagram of the mechanism of 2D material to enhance the reliability and comparison of AFM before and after passivation of defects [122] (copyright © 2017, Elsevier Ltd. All rights reserved); (**d**) schematic diagram of the thermal stress presetting and its counteracting mechanism (copyright © 2022, Elsevier B.V. All rights reserved).

Atomic Layer Infiltration (ALI) is also a derivative technology of ALD, as illustrated in Figure 25a. The principal characteristic of this technology is that the precursors and reactants of ALD are, in turn, infiltrated into the organic film. There are two distinct forms of material adsorption. On the one hand, the precursors and reactants are physically adsorbed in the interstitial space within the organic film; on the other hand, they may chemically bond with the organic bonds (e.g., C=O) within the film. The repeated infiltration of precursors and reactants results in the formation of a thin stratification of organic/inorganic mixed components at a certain depth (5~30 nm) on the surface of the organic layer. This layer exhibits an excellent water vapor barrier performance due to the filling of the voids in the organic bulk. The hybrid material also enhances the flexibility of ALI films. Seung Hun Kim et al. used TMA and H_2_O to form a 7~22 nm Al_2_O_3_/PI hybrid thin layer on a PI substrate [135]. This demonstrated durability when placed in an environment of 85 °C/85% R.H. for 1000 h and maintained a comparable WVTR value even after R1 mm radius bending, indicating a promising potential for practical application.

PMMA (acrylic) is the most common material for organic buffer layers utilized in the commercial manufacturing of TFEs. However, relatively little research has been conducted on this type of material for encapsulation. Byoung-Hwa Kwon et al. [95] demonstrated for the first time the feasibility of ALD-Al_2_O_3_ engineering applications using the structure of ALD-Al_2_O_3_/PMMA/ALD-Al_2_O_3_. The OLED large-size display encapsulated with this structure demonstrated no failure phenomenon under bending of R3.2 mm and 60 °C/85% R.H. environment for 305 h. Furthermore, the green OLED device encapsulated with this structure has a higher current efficiency (CE), which provides strong support for the value of the engineering application of the ALD-Al_2_O_3_ barrier film. Guixiong Che et al. [139] conducted a plasma fluorination treatment of the PMMA organic layer within the ALD-Al_2_O_3_/PMMA TFE structure. Following the SF_6_ plasma treatment of the organic layer, which resulted in the formation of an organic layer with a reduced WVTR and irregular surface spikes, as illustrated in Figure 25b, the TFE structure exhibited enhanced water vapor/oxygen barrier performance and improved bending reliability. It is hypothesized that the mechanism of fluorination treatment to enhance mechanical reliability can be attributed to the following factors: (a) The uneven surface morphology elongates the length of Al_2_O_3_ in the cross-section direction, which, along with the stress buffering effect of an organic film, enables the Al_2_O_3_ to release the stress into the organic film when it is subjected to tensile stress, thus raising the tensile critical strain. (b) The Young’s modulus of the organic surface increases, and thus the thermal expansion decreases after the treatment. For instance, after high-temperature deposition, the thermal stress between ALD-Al_2_O_3_ and the organic layer can be reduced, thereby reducing the internal stress of the encapsulation system and improving the mechanical strength. The equipment required for the plasma treatment is the same as that required for thin-film deposition used for commercialized barrier layers (SiN_x_, SiO_x_N_y_), which makes this technique cost-effective and highly replicable.

Two-dimensional materials are widely employed in electronic devices. Theoretically, the atomic spacing of graphene, graphene oxide, and hexagonal boron nitride (h-BN) is smaller than that of water and oxygen molecules, which can provide an effective barrier capacity. However, two-dimensional films prepared by conventional low-temperature equipment frequently display a high defect density, resulting in a markedly inferior barrier performance. Based on the aforementioned rationale, the combination of conventional barrier films and ultra-thin two-dimensional materials represents a crucial strategy for achieving effective encapsulation. As illustrated in Figure 25c, Dong-won Choi and Taewook Nam et al. [122,140] were able to significantly enhance the barrier performance of graphene by filling its defects using ALD-Al_2_O_3_. In a bending test, even if the ALD film produces cracks under stress, the 2D material at the cracks without defects can still provide barrier capacity, thus enhancing mechanical reliability. Wonseok Jang et al. [137] employed a similar approach to passivate h-BN flakes, resulting in an ALD-Al_2_O_3_ encapsulated h-BN film that can withstand 4% tensile strain, a notable degree of flexibility among novel TFE techniques. However, the current WVTR of this film is relatively low, and the preparation process is still under development, necessitating further research to fully ascertain its potential applications in TFEs.

#### 3.2.3. Stress Management Technology

The neutral plane is a region that exhibits zero stress during OLED bending. By adjusting the thickness and Young’s modulus of the organic material, adding a top organic layer [130], and adjusting the stacking structure of the entire OLEDs [141], the TFE layers can be placed in the neutral plane, thus enabling them to achieve an excellent mechanical reliability.

The application of a neutral plane represents a commonly employed technique within the domain of stress modulation. In contrast, Yong Cheon Park et al. [93] proposed the presetting of residual thermal stress in the direction of compression for ALD-Al_2_O_3_/organic stacked structures by modulating the thickness, Young’s modulus, and coefficient of thermal expansion of the organic buffer layer. This resulted in a counteracting effect of the encapsulation structure when subjected to tensile stress. As shown in Figure 25d, the authors achieved a structure that still performed a nearly unchanged WVTR under tensile strain of 1.09%. Specifically, the structure comprised only four layers of 10 nm Al_2_O_3_ and four layers of 200 nm p(CHA-co-V3D3) organic film, and the initial WVTR was 3.1 × 10^−5^ g m^−2^ day^−1^. The high transmittance of this encapsulation structure enabled no influence on the efficiency and lifetime of the OLEDs. However, the authors have not demonstrated the reliability of the structure in the harsh environment, which may be required for the engineering verification. Furthermore, it may be better to replace the ALD-Al_2_O_3_ film with a nanolaminate film, which can withstand stronger water vapor corrosion and further achieve ultra-flexibility through stress modulation.

As can be seen from Table 1 and Table 2, the thinning and ultra-flexibility technology have been studied a lot. However, the information from the two tables is massive, which can be comprehensively reviewed in terms of the two technologies. Table 3 is summarized for a brief review of different designs of TFEs to achieve thinning and ultra-flexibility technologies. For instance, the table was supplemented with subjective opinions and approximate values, which shall be regarded as a simple reference.

### 3.3. Multifunctionality Technology

The commercialization competition for OLED displays is intensifying, and the products are developing in the direction of portability and diversification. This includes the emergence of wearable displays and vehicle displays. The production of lower-cost, thinner, and more performant OLED displays will constitute an important development direction for OLED display technology in the future. As an organic electroluminescent device, the operational characteristics of OLED are dependent on a variety of physical processes, including the transportation of electrons and holes, the generation of luminescence and heat from exciton pairs, and the propagation of light. In addition to the sensitivity to water and oxygen that stems from the organic composition of the material, other factors such as carrier transport characteristics, luminescence and heating properties, and the light extraction rate also have an influence on the performance of OLED displays. This section aims to present a review of how TFEs can fulfill multiple roles simultaneously, acting not only as a barrier layer but also as a multifunctional component capable of enhancing display products through their innovative integration.

In the context of top-emitting OLED devices, a sufficiently high visible light transmittance represents a fundamental prerequisite for TFEs. Given that light must traverse the TFE structure, particularly when the microcavity effect of the OLED device is minimal, the refractive index and thickness configurations of the encapsulation film will also influence the light extraction efficiency of the OLEDs. The use of optical simulation and similar tools to adjust the refractive index and thickness of the multilayer encapsulation film in order to maximize the light efficiency of the OLEDs represents one aspect of achieving the multifunctionality of TFEs. Furthermore, researchers can also design the TFE to cooperate with the entire OLED device multidimensionally through targeted optical engineering, which can lead to targeted optical effects.

**Figure 26:**
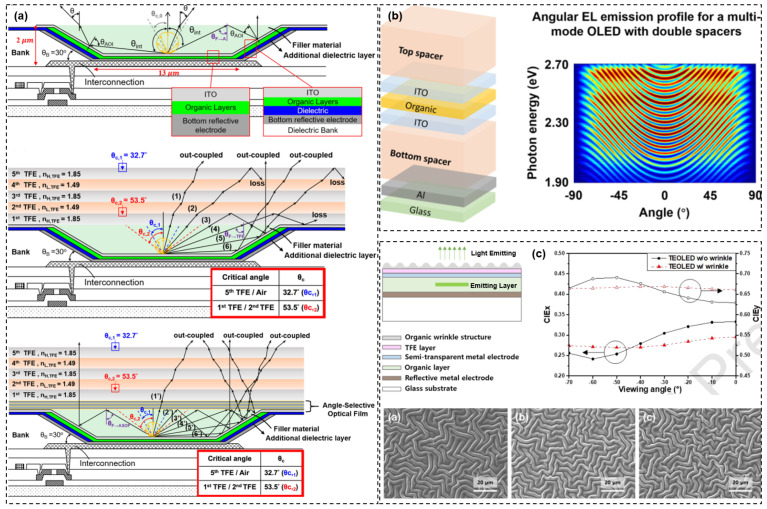
Schematic application of (**a**) angle-selectable layer, (**b**) optical support layer (copyright © 2020, American Chemical Society), and (**c**) wrinkle layer (copyright © 2019, The Korean Society of Industrial and Engineering Chemistry, Published by Elsevier B.V. All rights reserved).

Po-Hsiang Liao et al. [142] examined the impact of a display pixel structure comprising a cup-shaped reflector and a high-index filler layer on the optical efficiency of OLEDs. As shown in Figure 26a, the introduction of TFEs was demonstrated to exert a significant influence on the light output efficiency. This is due to the fact that the TFE structure, comprising alternating high/low-index laminates, results in a relatively small total reflectance angle for the light emitted from the OLEDs. Consequently, the light is prone to refraction and consumption within the device. By incorporating an angle-selectable optical stack between the TFEs and the OLEDs, the authors were able to restrict a proportion of the light with an angle exceeding the total reflection angle to be refracted back from the optical stacks and subsequently converted to light with an angle less than the total reflection angle, which was then emitted after mirror reflection by the slopes of the reflectors. This resulted in a greater proportion of light being extracted.

In order to alleviate the brightness and chromaticity deviation of OLEDs at large angles, as shown in Figure 26b, Dong Chen et al. [143] introduced two optical support layers (Spacer), which are added at the upper and lower ends of the OLED device. This played the role of removing the microcavity effect of the top-emitting OLED device and realizing the multimode light output, so that the light’s dependence on the angle became very weak, which substantially enhanced the chromaticity and luminance viewing angle of OLEDs. Moreover, this effect is appropriate to the full wavelength of optical light, which can effectively enhance the color performance of OLEDs in the bending deformation part.

Sangsoo Jang et al. [144] enhanced the light-outgoing angle of OLEDs by adding a winkle structure to the TFE, as shown in Figure 26c. The surface of this winkle structure exhibits an uneven morphology, which can effectively distribute the light. Compared to another optical functional structure called microlens, it did not lead to the issue of image ghosting, and the preparation method is relatively simple, which is of great value for engineering applications.

From an optical perspective, in addition to incorporating auxiliary film and modifying its structure to serve a particular function, the formation of a Distributed Bragg Reflector can be achieved by adjusting the refractive index and the thickness of the TFE layers. The DBR is capable of blocking a specific wavelength of light. In the case of OLED displays, while this blocked light is UV light, the DBR can effectively prevent UV light from damaging the organic material, thus improving efficiency and lifetime. The precise film thickness control capability of ALD films allows TFEs to realize both UV light blocking and excellent barrier performance.

**Figure 27:**
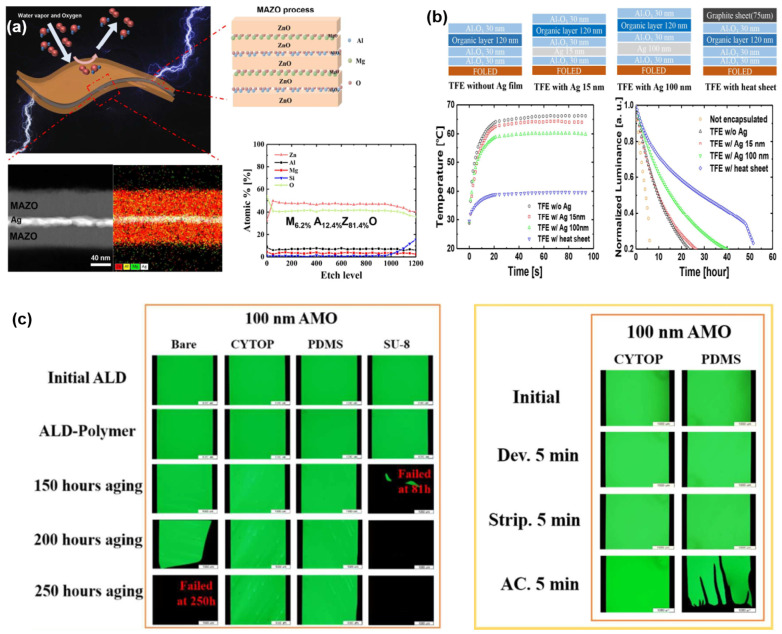
Multifunctionalities of TFEs in terms of (**a**,**b**) electric and (**c**) chemical tolerance (copyright © 2018, American Chemical Society; © 2017, American Chemical Society; and © 2013, Royal Society of Chemistry, respectively).

From the electrical point of view, since the materials of TFE are basically metal oxides, metal nitrides, and their multi-compounds, they can be used as dielectric layers at the same time. The dielectric/metal/dielectric (DMD) structure can be well realized for the multifunctionalities of TFEs. As shown in Figure 27a, Hyun Kwon Jeong et al. [145] successfully realized the multifunctionalities, performing both electrode and encapsulation with the MAZO/Ag/MAZO structure. Specifically, MAZO is a Mg- and Al-doped ZnO multi-compound film, and by adjusting their ratios, the DMD structure can realize low resistance and high transmittance while ensuring the encapsulation performance. Moreover, the authors formed the DMD structure by inserting an Ag film into the structure; as shown in Figure 27b, it can serve as an encapsulation while dissipating heat and lowering the OLED operating temperature, thus enhancing the OLED display lifetime [22]. However, the thickness of the Ag film cannot be too thick under the condition of ensuring the optical transmittance, so the actual heat dissipation effect was not outstanding. The effect of adding a graphene heat dissipation film directly on TFE will be better.

From the perspective of the OLED fabrication procedure, after the completion of TFEs, some display products would still need to form a touch panel on TFEs, which can reduce the thickness of the overall OLED module structure to enhance the flexibility. The fabrication of touch panels requires that the TFE be able to tolerate solvents such as developer (Dev.), stripper (Strip.), and acetone (AC.) used in the lithography process. As shown in Figure 27c, Lei Wang et al. [48] investigated the tolerance performance of organic protective films with different materials coated on ALD-deposited AMO films (nanolaminates of Al_2_O_3_ and MgO). The results showed that the combination of AMO + CYTOP can effectively resist chemical solutions while improving the stability of AMO in high-temperature and high-humidity environments (no failure for 250 h in 85 °C/85% R.H.).

### 3.4. Emerging Technology

The contemporary era is witnessing a period of accelerated growth for flexible electronic products. The traditional hard shell encapsulating the terminal of electronic devices has been replaced by flexible and even implantable alternatives. The emergence of new fields of flexible electronic technology and materials, including healthcare applications [146,147], human sports detection [148], and electronic skin [149,150], has led to the rise in novel demands. To fulfill the potential of these emerging domains, the next generation of flexible display technology will likely encompass stretchable, fabric, and implantable displays.

**Figure 28:**
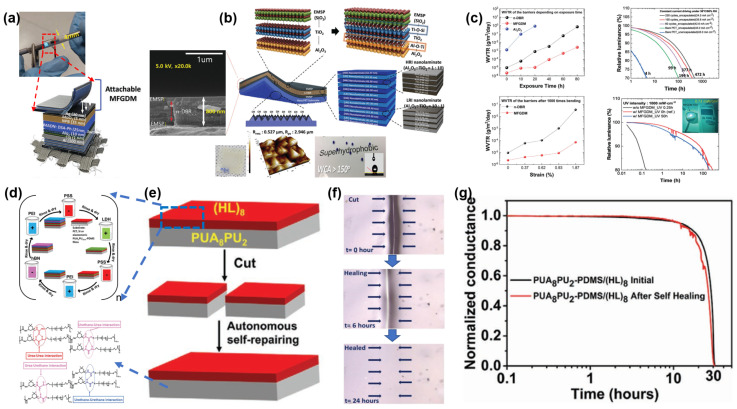
(**a**,**b**) Schematic diagrams of the composition of multifunctional gas diffusion multibarrier and the structure of the fabric display; (**c**) illustration of the encapsulation of the fabric display and the performance of the UV light attenuation; (**d**,**e**) schematic diagrams of the preparation and working principle of the self-healing barrier; (**f**,**g**) illustration of the repair performance of the self-healing barrier (copyright © 2023, Wiley-VCH GmbH).

A cloth would be subjected to a series of processes during its use, including pulling, folding, storing, washing, and drying. Therefore, displays applied to the cloth of fabrics must be waterproof and watertight, as well as able to be bent and folded at will, and resistant to ultraviolet rays. Yongmin Jeon et al. [151] successfully fabricated fabric displays on fabrics with a bending radius of down to 4 mm that can be immersed in water by superimposing a series of emerging technologies, as illustrated in Figure 28a,b. In particular, the author initially obtains PET with a serrated surface morphology through ion beam bombardment and then forms a layer of fluor-octyl trichlorosilane (FOTS) with hydrophobic properties on these serrated microstructures, thereby creating a superhydrophobic PET substrate. Subsequently, a 150 nm organic film comprising SiO_2_ particles is deposited on the substrate. The ALD Al_2_O_3_/TiO_2_ nanolaminate, which exhibits UV-blocking properties, is then sandwiched between the organic films, forming an encapsulation structure with high barrier properties and high bending reliability. In contrast to conventional encapsulation structures, such as inorganic/organic/inorganic, the authors discovered that organic/inorganic/organic structures are capable of exhibiting a critical strain that is more than double that of conventional ones. Furthermore, the formation of more dense Ti-O-Si and Al-O-Ti bonds at the SiO_2_/organic layer, Al_2_O_3_/nanolaminate, and TiO_2_ film interfaces enables the encapsulation structure to achieve a WVTR level of 10^−6^ at a relatively low temperature of 60 °C. The fabrication of superhydrophobic materials and microstructures, the establishment of ALD nanolaminates, the optimization of emerging organic materials and structures, and the combination of these emerging technologies have formed a synergistic effect. This structure, which the authors have named the multifunctional gas diffusion multibarrier (MFGDM), is illustrated in Figure 28c. Following an RA test conducted in an environment of 85 °C/85% RH for 80 h, the WVTR of the MFGDM structure decreased to a level of 10^−3^ g m^−2^ day^−1^. In comparison, the structure comprising solely of ALD nanolaminates exhibited a significant deterioration, reaching a WVTR level of 10^−1^ g m^−2^ day^−1^. Furthermore, the WVTR exhibited a deterioration of only one order of magnitude at a strain of 1.87% during the bending test. Furthermore, the microstructured PET substrate enhances light efficiency, thereby improving the lifetime of the material. Finally, the UV-blocking ALD Bragg distributed nanolaminates permit the outdoor exposure of fabric displays without the rapid darkening that would otherwise occur.

From an engineering perspective, the cost of the ALD process remains a significant obstacle to the implementation of the aforementioned technique. More importantly, the authors employed a bonding process to integrate the MFGDM and OLEDs. If the barrier performance of the adhesive is inadequate, it could potentially impede the reliability factor. Furthermore, the fabrication of a fabric display remains a highly immature process. For instance, the formation of an effective barrier film on the fabric, the expansion to large-scale manufacturing, flexible substrate handling, and bottlenecks due to low-temperature processes are all obstacles that require further investigation and resolution.

As an additional emerging flexible display product, during the stretching operation, the stretchable display would be subjected to tensile strain on the bulk of the display in order to generate stretching deformation. The extent of strain required for different products varies. Currently, the critical strain that TFEs can withstand is less than 2%, which is insufficient for stretchable flexible displays. Accordingly, self-healing techniques would be a preferred encapsulation method, as they can repair cracks caused by strain [152]. Mahmood et al. [153] developed a self-healing barrier film for encapsulation. The self-healing barrier film consists of two main parts, as shown in Figure 28d,e, which are (HL)8 with barrier capability and PUA8 PU2 -PDMS with self-healing capability, respectively. The barrier capability of (HL)8 is attributed to the water vapor path lengthening effect of the stacked 2D sheet material [154]. And, the source of the self-healing capability of PUA8 PU2 -PDMS is the hydrogen bonding connection between urea and urethane functional groups within the material. As shown in Figure 28f,g, the surface morphology after repair was intact, and the WVTR before/after repair was basically unchanged.

**Figure 29:**
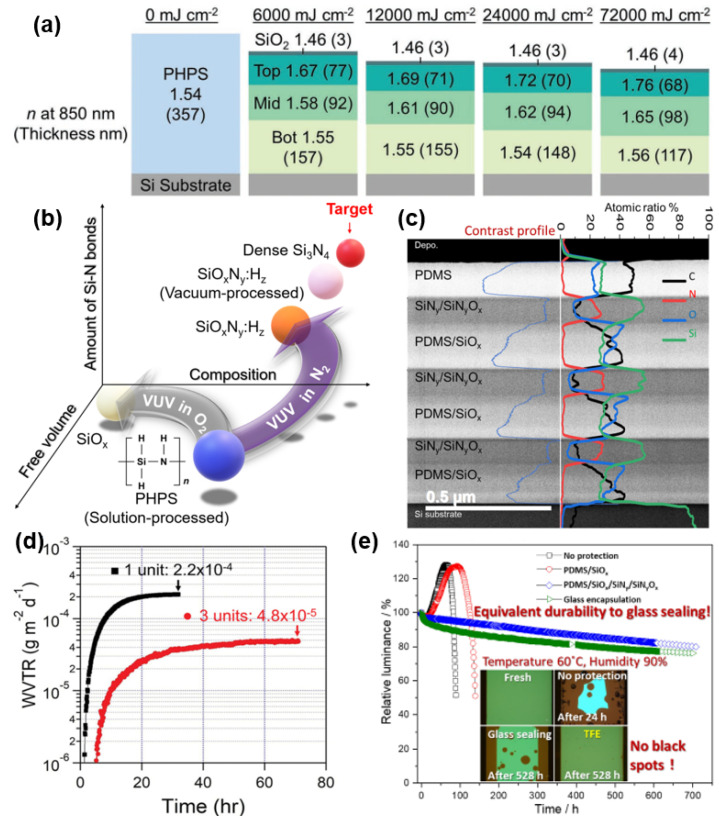
(**a**–**c**) Schematic diagrams of the fabrication and mechanism of PONT and (**d**,**e**) illustrations showing its encapsulation performance.

The large-size OLED display is still in the engineering verification stage, and the production cost is the most significant concern for enterprises. The current TFEs of small-size OLED displays utilize PECVD to complete the deposition of the barrier film. As vacuum equipment, it is costly and requires a substantial investment in maintenance. If the barrier film could be deposited using a wet solution, it would represent a significant advancement in reducing the cost of OLED manufacturing. Tatsuki Sasaki et al. [59,155,156] used a full solution process to obtain a PONT (polymer/oxide/nitride/ternary) barrier structure with a high barrier performance, as shown in Figure 29. The WVTR of three dyads of PONT was found to be 4.8 × 10^−5^ g m^−2^ day^−1^. Specifically, the PONT is composed of a combination of two distinct films: a PDMS/SiO_x_ film, prepared by vacuum ultraviolet (VUV, 172 nm) irradiation on PDMS (polydimethylsiloxane) in an oxygen-enriched environment, and a SiN_y_/ SiO_x_N_y_ film, prepared by the same irradiation on PHPS (perhydropolysilazane) in a nitrogen-enriched environment. The PDMS/SiO_x_ film could protect the OLEDs from being damaged by the solvent of PHPS, because the D5 (decamethylcyclopentasiloxane) solvent used in the coating process of the PDMS was verified not to affect the performance of the OLED device. The PDMS/SiO_x_ film comprises large free volumes, thus a low water vapor barrier. In contrast, the SiN_y_/SiO_x_N_y_ layer is the main barrier layer. The film thickness reduction after VUV irradiation was extremely obvious, and the shrinkage was strong, which proved that PHPS was transformed into the denser SiN_x_/SiO_x_N_y_. Since VUV is radiated from the top to the bottom, it provides a mechanism for stress buffering in the transformation of PHPS to SiN_x_/SiO_x_N_y_. It was verified that the OLED display using the PONT encapsulation structure did not fail even after 528 h of storage in an environment of 60 °C/90% R.H. Moreover, the emitting efficiency and lifetime of PONT-encapsulated OLEDs were comparable to those of glass-encapsulated OLEDs, which is very promising for practical application. In a similar manner to the utilization of thin films fabricated by a solution process as an alternative to PECVD, Green et al. [157] proposed a method for fabricating ALD oxide films using a solution process. Furthermore, the authors deposited the films onto the shell of individual QDs with the objective of enhancing their barrier properties. Although the authors did not perform an evaluation of the barrier performance, as a next-generation light-emitting layer for OLED displays, the method of water vapor barriers for QDs themselves may be a promising direction to be studied.

### 3.5. Novel Equipment for Thin-Film Encapsulation

As outlined in Section 3.1, although ALD films have demonstrated robust barrier properties and flexibility, their slow deposition rates and high equipment maintenance costs have resulted in delays in their utilization in commercialized products. However, simultaneous advancements in equipment and processes may potentially offset these shortcomings. This section will introduce some novel equipment technologies and attempt to assess their engineering feasibility.

**Figure 30:**
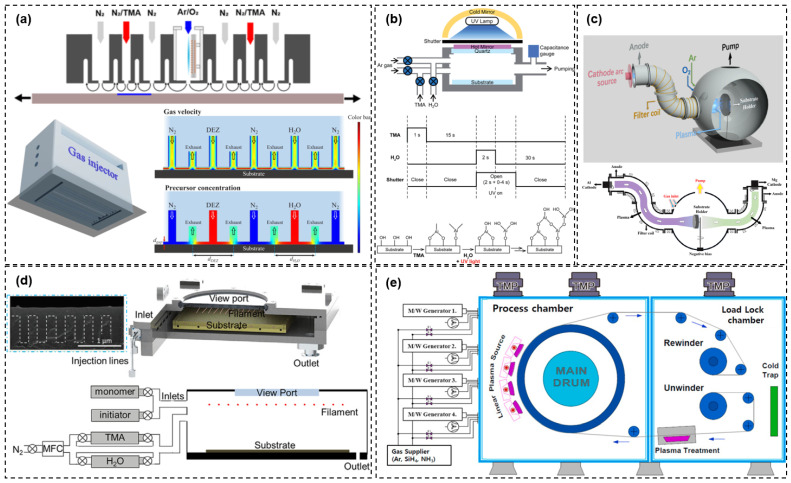
Schematic diagrams of apparatus including (**a**) spatial ALD [43] (copyright © 2017, American Chemical Society) [160], (**b**) UV-ALD (copyright © 2021, Elsevier B.V. All rights reserved), (**c**) FCVA (copyright © 2022, Elsevier Ltd. All rights reserved), (**d**) iCVD + ALD (copyright © 2022, Wiley-VCH GmbH), and (**e**) R2R (copyright © 2021, Published by Elsevier Ltd.).

Spatial ALD (SALD) is regarded as the most promising equipment for addressing the limitation of the slow deposition rate associated with time-divided ALD [158]. As illustrated in Figure 30a, the apparatus delivers precursor and reaction gases in a separate manner through a gas nozzle, with vacuum extraction ports and N_2_ gas curtains situated around the nozzle. When the distance between the nozzle and the substrate is sufficiently narrow, the precursor and reaction gases are in contact with the substrate in disparate locations, preventing interference between the two. Subsequently, the substrate is successively contacted with the precursor and the reactive gas in accordance with the movement of the substrate or nozzles, thereby completing one ALD cycle and enabling the completion of multiple cycles through reciprocating motion. A number of studies have demonstrated that the barrier capacity of SALD-prepared films is comparable to that of temporal ALD [43,85,159,160,161,162]. Furthermore, SALD can fabricate multi-compound films similarly to temporal ALD. Additionally, SALD can be operated in an atmospheric environment to achieve thin-film deposition. The slow deposition rate of temporal ALD was addressed by SALD from a fundamental standpoint, offering significant engineering value through the conversion of time costs into spatial costs. However, when considering the expansion of SALD to large-scale manufacturing, several issues still require resolution.

(a) The introduction of the substrate motion strategy results in a greater occupied space, exceeding that of the temporal ALD by a factor of two.
(b) The reaction’s inherent limitations necessitate the control of substrate/nozzle movement speed (less than 20 mm/s). At this juncture, the requisite time for the fabrication of a circulating ALD film on the G8.5 glass substrate (2200 × 2500 mm) is a minimum of 110 s. The completion of a 30 nm Al_2_O_3_ film would require approximately six hours, rendering the process no faster than that of temporal ALD. Although this situation can be achieved by adding multiple groups of nozzles to complete multiple ALD cycles in a single reciprocating motion, this approach also greatly increases the procurement and maintenance costs of the equipment.
(c) When it is necessary to complete one ALD cycle with one reciprocation, the length of the nozzle should be set to the width of the large-size substrate. Thus, the control of the uniformity of the nozzle’s gas ejection in the longitudinal direction is a key factor affecting the uniformity of the depositing ALD film. This has elevated the requirements of the fluid design.
(d) The current small-size TFEs use a mask containing a metal sheet to realize the film patterning. The mask sheet is directly covered on the substrate. Only the opening area can deposit the thin film. If the patterning method is directly transplanted, the mask sheet may bend, scratch, or otherwise fail to function properly. The substrate-moving platform must also be highly load-bearing and stable, which presents a significant challenge for SALD equipment developers. As a result, effective patterning methods remain elusive. The strategy of area-selective ALD has been developed in recent years as a patterning method [163,164]. However, additional manufacturing steps and appropriate equipment design still remain to be further studied and verified by engineers.

Kwan Hyuck Yoon et al. [165] developed UV-ALD equipment based on ALD, as shown in Figure 30b. The introduction of UV irradiation at the H_2_O purge and reaction stage was realized by making a transparent quartz roof above the reaction chamber, which could significantly reduce the -OH bond content inside the film and increase the density of the deposited Al_2_O_3_ film, and a WVTR of 9.2 × 10^−7^ g m^−2^ day^−1^ was achieved. The structure of this equipment is simple, and the manufacturing cost is relatively low, but there may be the problem that it is difficult to expand to large-scale equipment. The main reasons are as follows: (a) the quartz glass above the large size state will be cracked due to excessive vacuum pressure; (b) the process only needs UV irradiation in the reaction stage, and the UV lamp is blocked by the shutter in other stages, so the mechanical movement cannot be quickly switched in the large-scale equipment, which will seriously affect the margin of the process, and compressed the room for improvement in the efficiency of the process. Speculatively, the timing of the UV irradiation stages and the upgrading of the equipment design still need to be further developed.

Yuan et al. [166,167,168] employed the magnetic Filtered Cathode Vacuum Arc (FCVA) deposition technique for the deposition of TFEs. As illustrated in Figure 30c, the apparatus primarily comprises a deposition chamber, a magnetic filtering elbow, and a cathodic arc generator. During the deposition of Al_2_O_3_, the cathodic arc generator initially utilizes the aluminum target as the cathode, generating a moving cathodic discharge arc on the aluminum target. This arc sputters the target and produces a multitude of particles, which may contain uncharged cluster particles, aluminum ions, electrons, and other constituents. The Lorentz force permits ions and electrons in the elbow to alter their direction of travel and reach the mouth of the elbow, thereby filtering the majority of impurity particles. Consequently, only the ions and electrons are able to reach the target deposition substrate. Subsequently, a thin film may be formed by the accumulation of ions, which is why this deposition technology is also known as ion beam deposition. The films formed by ion beam deposition are characterized by a dense structure and a low level of impurity defects due to the high binding force and high energy state of the ions. The authors conducted the deposition of Al_2_O_3_ films by introducing O_2_ and Ar into the chamber. The WVTR of the ~100 nm Al_2_O_3_ film grown by FCVA deposition was determined to be 9.9 × 10^−4^ g m^−2^ day^−1^. The prepared Al_2_O_3_ exhibited high transmittance and displayed minimal destructive effects on organic materials. One of the key benefits of the FCVA over ALD is the ability to regulate the deposition rate through the manipulation of the ion beam density, which can be increased to a level up to 1.5 to 4 times higher than that of the typical ALD deposition process. Furthermore, the deposition process does not result in the formation of additional impurities, thereby ensuring the material’s high purity. The concentration of carbon and hydrogen is notably lower than that observed in ALD. Nevertheless, further properties need to be verified, such as intrinsic stress and compatibility.

Bong Jun Kim et al. [124] developed the iCVD equipment based on ALD equipment, as illustrated in Figure 30d. Subsequently, it is possible to deposit ALD (inorganic)/iCVD (organic) laminated films in the same chamber. The combination of iCVD and ALD equipment is achieved by incorporating a heating filament within the chamber, which enables the polymerization reaction of organic monomers and the deposition of the film. The iCVD process offers several advantages, including the following:

(a) Low-temperature deposition (10~60 °C);
(b) The ability to deposit films of high purity, low pinhole density, and good step coverage;
(c) The capacity to fill nanometer pinholes on the surface of inorganic films and provide a smooth deposition surface for inorganic film deposition.

The most significant strength of the iCVD process is its capacity to form a film through the combination of a diverse range of monomers, characterized by varying functional groups, rheological properties, and glass transition temperatures. Such processes can regulate mechanical parameters, including Young’s modulus, and impart a strong planarization performance to the film. By modifying the ratio of monomers, including GA, DMAEMA, and CHA, Yong Cheon Park et al. [90] developed an organic film with superior planarization properties, as illustrated in the insert diagram in Figure 30d, which significantly enhanced the reliability of encapsulation. Moreover, iCVD exhibits excellent capabilities in defect passivation and film thickness control, rendering it the preferred process for the thinning of TFEs. From an equipment perspective, iCVD equipment is relatively modest in its complexity, drawing upon the design experience of CVD equipment. Its scalability is not an obvious limitation. However, the replacement and maintenance of equipment parts, such as filaments and the particles generated, may become a disadvantage. As iCVD equipment technology has only recently been applied to TFEs, there is still a considerable distance to traverse before it can be considered for commercial application.

The Roll to Roll (R2R) process is the ultimate development direction for the fabrication of flexible electronic devices. As shown in Figure 30e, Seong-Keun Cho et al. [169] developed R2R microwave plasma-enhanced chemical vapor deposition equipment.The developed 100 nm SiN_x_ thin films can reach a WVTR of 7 × 10^−3^ g m^−2^ day^−1^. The authors achieved stable SiN_x_ thin films by controlling the ratio of gases, which pushed forward the preparation of thin films by the R2R process.

## 4. Summary and Outlook

The development of large-size flexible OLED displays necessitates the creation of novel TFE technologies that are more reliable and exhibit extreme flexibility. The large-scale manufacturing for commercial large-size flexible OLED displays has not been widely conducted, and to be exact, it is still under the engineering validation phase. There are many issues to solve while in the engineering validation phase, which may be neglected in the laboratory phase. For example, we propose that enhancing the passivation ability of TFE to particles and extending the encapsulation lifetime in the presence of particles would help improve product quality and reduce manufacturing costs in large-scale manufacturing. Moreover, the compatibility of TFE and OLEDs/TFTs would be a crucial aspect that needs to be validated. We believe that engineering reflects the thought of unification, integration, and verification. To ensure optimal engineering validation of TFE, it is essential to ascertain the ability to meet a multitude of specifications. These include, but are not limited to, the WVTR, stress, transparency, deposition rate, adhesion, compatibility with OLEDs/TFTs, being MURA-free, particle coverage, wet process tolerance, harsh environment endurance, and flexibility. In order to achieve engineering verification, this paper presents a review of the research progress that has been made in novel TFE technologies, including those that have been developed for thinning, ultra-flexibility, multifunctionality, novel equipment, and emerging technologies. However, it is acknowledged that further comprehensive verification and development are still required in order for these technologies to meet the requirements of engineering applications.

With regard to the TFE thinning technique, ALD is the most promising process technology. However, its deposition rate is relatively slow, and the potential for hydrolysis problems raises concerns about the reliability of the display screen. Furthermore, the high maintenance costs remain a challenge to be addressed. The development of a rapid ALD process, which combines the advantages of the fast deposition rate of PECVD with the thin thickness of ALD, may provide a direct and effective solution in engineering applications in terms of thinning technology. On the other hand, ALD nanolaminate represents a significant technological advancement in the field of thin barrier layers, offering a reliable solution for engineering applications. Comprehensive and systematic verification of this technology will facilitate the commercialization of OLED display products in high-reliability thinning technology.

In the context of ultra-flexibility technology for TFEs, the integration of ALD with organic films via a nanolaminate structure represents a highly promising approach with significant potential for engineering applications. A variety of organic materials have been shown to exhibit an excellent encapsulation and flexibility performance. However, given the current research progress, MLD still suffers from a low deposition rate, S-H nanocomposites lack a scale-up process technology, iCVD still requires further mechanical reliability verification, and some other techniques still require further environmental reliability verification.

In terms of the multifunctionalities of TFEs, TFEs that assist in improving the light-extraction efficiency of OLEDs have a very high value for engineering applications. Cooperation in the design of OLED devices via simulation and calculation, the introduction of multifunctional TFE films, and the enhancement of TFEs’ added value would constitute a highly meaningful route of technological development. The multifunctionality of TFEs in terms of optoelectronic and manufacturing innovations requires alignment with the demands of practical OLED products. The main objective of development in multifunctional TFEs is to achieve adaptability in material, process, and structure for different products.

In the emerging technology of TFEs, fabric display and intrinsic stretchable display are the primary drivers of development. However, such products are still in the laboratory development stage, and the process route of large-scale production may diverge significantly from the existing manufacturing system. Accordingly, further systematic process research is required.

In summary, the design of TFE materials, processes, and structures is a systematic and complex project. The engineering verification of basic characteristics, reliability, and compatibility in order to establish a TFE strategy that meets the requirements of highly reliable and ultra-flexible products will become a valuable contribution to the field of engineering.

## Figures and Tables

**Table 1:** The advancement of thinning technologies in accordance with engineering specifications.

Film Type	Fabrications							Basic Properties			Reliability		Compatibility Verify	Ref.
	Equipment	Material	Precursor	Dep. Temp. *(°C)	Thickness (nm)	GPC (nm/ cycle)	Dep. Rate * (nm/min)	WVTR (g m^−2^ day^−1^)	Test Evn. * (°C)/RH (%)	Transparency (Visible Spectrum)	Accelerating Aging Test	Bending Test		
Inorganic laminate	ALD/ CVD	Al_2_O_3_/SiN_×_	TMA/H_2_O SiH_4_/N_2_O/NH_3_	RT/80	20/200	×	×	5.80 × 10^−2^	38/100	×	312 h * OK @25/80	×	×	[81]
	PECVD/ ALD	Al_2_O_3_/SiN_×_		30/100	205 (160/45)	×	×	7.00 × 10^−3^	38/90	×	×	×	×	[89]
		Al_2_O_3_/SiO_×_				×	×	2.40 × 10^−4^	38/90	×	×	×	×	[89]
Monolayer	ALD	Al_2_O_3_	TMA/H_2_O	80	50	×	×	1.00 × 10^−1^	38/100	>85.0%	×	×	Same CE *	[81]
			TMA/H_2_O	80	100	×	×	1.40 × 10^−2^	25/80	×	×	×	×	[97]
			TMA/O_3_	80	100	×	×	4.00 × 10^−3^	25/80	×	×	×	×	[97]
			TMA/H_2_O	120	26	×	×	1.10 × 10^−3^	23/50	×	×	~1.0 × 10^−3^ @R20 mm 5cyc		[98]
			TMA/O_3_	100	100	×	×	7.05 × 10^−4^	50/50	≈95.0%	×	×	×	[99]
			TMA/H_2_O	80	60	0.090	0.135	4.90 × 10^−4^	20/60	×	×	×	Same CE Half lower LT *	[100]
			TMA/H_2_O	80	73	0.083	0.083	2.10 × 10^−4^	25/80	×	×	×	Higher CE	[67]
			TMA/O_3_/H_2_O	80	47	0.094	×	5.43 × 10^−5^	40/100	×	160 h OK @40/100	×	×	[101]
			TMA/O_3_	80	81	0.092	0.138	8.70 × 10^−6^	25/80	×	×	×	Higher CE	[67]
			TMA/O_3_	80	60	0.090	0.268	8.70 × 10^−6^	20/60	×	×	×	Same CE Same LT	[100]
		MgO	Mg (CpEt)_2_/H_2_O	70	60	×	×	5.83 × 10^−2^	30/90	84.0%	×	×	×	[102]
		ZrO_2_	TDMAZr/O_3_	100	100	0.095	×	3.87 × 10^−3^	50/50	≈85.0%	×	×	×	[99]
			TDMAZr/H_2_O	80	80	×	×	3.74 × 10^−3^	20/60	×	×	×	Worse LT	[103]
			TDMAZr/O_3_	80	80	×	×	6.09 × 10^−4^	20/60	×	×	×	Same LT	[103]
	PEALD (local)	Al_2_O_3_	TMA/O_2__PLS *	100	50	0.180	×	3.75 × 10^−4^	60/90	×	3000 h peeling @40/90	×	Inferior LT (OTFT)	[104]
		TiO_2_	TDMAT/O_2__PLS	100	50	0.075	×	6.32 × 10^−4^	60/90	×	×	×	×	[104]
	PEALD (remote)	Al_2_O_3_	TMA/O_2__PLS	100	100	×	×	9.50 × 10^−3^	50/50	>95.0%	×	×	×	[105]
		SiN_×_	SiH2(NHtBu)_2_/N_2__PLS	120	10	×	×	1.00 × 10^−6^	20/50	×	53 days NG @20/50 40 nm	×	×	[75]
		ZrO_2_	TEMAZr/O_2__PLS	100	100	×	×	1.09 × 10^−2^	50/50	<85.0%	×	×	×	[105]
	Spatial ALD (atmosphere)	Al2O3	TMA/H_2_O	75	100	0.180	×	6.00 × 10^−3^	60/60	×	×	×	×	[83]
			TMA/O_2__PLS	75	100	0.170	×	7.00 × 10^−4^	60/60	×	×	Same WVTR @R20 mm	×	[43]
			TMA/O_2__PLS	100	50	0.110	×	3.00 × 10^−4^	50/50	×	×	×	×	[43]
			TMA/O_2__PLS	100	100	×	×	2.00 × 10^−4^	60/60	×	×	×	×	[85]
			TMA/H_2_O	150	100	0.150	×	8.00 × 10^−5^	60/60	×	×	×	×	[43]
			TMA/O_2__PLS	150	100	0.110	×	5.00 × 10^−5^	60/60	×	×	×	×	[43]
			TMA/H_2_O	100	50	0.150	×	2.00 × 10^−5^	50/50	×	×	×	×	[43]
			TMA/O_3_	100	50	0.160	×	2.00 × 10^−5^	50/50	×	×	×	×	[85]
	PECVD	H: SiON	SiH_4_/N_2_O/NH_3_/H2	100	80	×	43.0	5.00 × 10^−5^	38/100	81.4%	720 h OK @RT	5 × 10^−1^ @ R 3 mm 3000 cyc	Higher CE	[85]
Nanolaminate	ALD	Al_2_O_3_/ZrO_2_	TMA/TDMAZr/O_3_	100	25/25	×	×	4.21 × 10^−4^	50/50	≈90.0%	×	×	×	[99]
			TMA/TDMAZr/O_3_	100	10/10	×	×	3.97 × 10^−4^	50/50	≈90.0%	×	×	×	[99]
			TMA/TDMAZr/O_3_	100	1:1cyc	×	×	3.26 × 10^−4^	50/50	≈90.0%	×	×	×	[99]
			TMA/TDMAZr/H_2_O	80	20 (2.1/3.1)	×	×	3.20 × 10^−4^	80/80	×	×	×	×	[106]
			TMA/TEMAZr/H_2_O	80	30(0.5:1.5)	×	×	2.00 × 10^−4^	85/85	×	300 h OK @100 nm 85/85	×	×	[107]
			TMA/TDMAZr/H_2_O	80	20 (2.6/3.6)	×	×	4.70 × 10^−5^	70/70	×	×	×	Same CE Inferior LT	[108,109]
		HfO_2_/ZnO	TDMAHf/DEZn/H_2_O	150	181 (1:19)	0.170	×	6.30 × 10^−6^	N/D	>85.0%	×	×	×	[110]
		Al2O_3_/SiO_×_	tris-(tert-pento×y) silanol/TMA/H_2_O	175	86	×	×	5.00 × 10^−5^	38/100	×	×	×	×	[72]
	PEALD (local)	Al_2_O_3_/TiO_2_	TMA/TDMAT/O_2__PLS	100	50	0.255	0.339	1.81 × 10^−4^	60/90	×	3000 h OK @40/90	×	Same LT (OTFT)	[104]
			TMA/TDMAT/O_2__PLS	100	49.8	0.405	0.485	9.16 × 10^−5^	60/90	77.0%	209 h @60/90	×	Inferior CE	[28]
	PEALD (remote)	Al_2_O_3_/ZrO_2_	TMA/TEMAZr/O_2__PLS	100	25/25	×	×	6.70 × 10^−3^	50/50	89.0%	×	×	×	[105]
				100	10/10	×	×	2.70 × 10^−3^	50/50		×	×	×	[105]
				100	5/5	×	×	1.30 × 10^−3^	50/50		×	×	×	[105]
				100	2/2	×	×	1.20 × 10^−3^	50/50		×	×	×	[105]
				100	100	×	×	9.90 × 10^−4^	50/50		×	×	×	[105]
2D	CVD	Graphene	N/A	RT	6 layer	×	×	1.78 × 10^−2^	25/45	85.5%	×	×	Little higher CE	[111]

* PLS = plasma; Dep. Temp. = deposition temperature; Dep Rate = deposition rate; Test Evn. = test environment; h = hours; CE = current efficiency; LT = lifetime.

**Table 2:** The advancement of ultra-flexibility technologies in accordance with engineering specifications.

Encapsulation Structure								Basic Properties			Bending Test			Reliability	Compatibility Verify	Ref.
Inorganic Layer				Organic Layer			Total Thickness/nm	Stress /MPa	Transparency	WVTR (g m^−2^ day^−1^)	WVTR or Failure Status (g m^−2^ day^−1^)	Strain (T = Tensile, C = Compressive)	Radius/mm	Accelerating Aging Test		
Equipment	Material	Dep. Temp. */°C	Thickness/nm	Equipment	Material	Thickness/nm				Before Strain	After Strain					
ALD	Al_2_O_3_	100	25–50	CVD	Graphene	×	×	×	<2% to Al_2_O_3_	2.62 × 10^−4^	7.65 × 10^−4^	0.89%	7	×	×	[122]
		100	5	Evaporation	4-BP	N/A	5 × 6	×	×	×	No crack	×	1	2.5 h OK @85/85	×	[123]
		90	25	iCVD *	pV3D3	100	100/25 × 6dyads	×	>90% (with glass)	8.10 × 10^−5^	Maintain barrier property	×	25	720 h OK @85/85	Same CE	[124]
		90	10		p(CHA-co-V3D3)	200	10/200/10/200	22.5 (1dyad)	99.70%	3.10 × 10^−5^		1.09% (T)	2.3	×	Inferior CE Same LT	[93]
		90	60	Inkjet	PMMA+Fluoridation	7000	60/7000/60/7000/60/7000	×	70% @3 dyads 90% with one more PMMA	1.02 × 10^−6^	Little cracks	(T)	3	≈36 h OK @60/85	Little higher LT	[93]
		90	15	MLD *	Alucone	2.5	102.5	×	≈95%	1.10 × 10^−4^	Little damage	0.72%	12	≈100 h @25/60	×	[125]
		80	10.4		SAOLs (7-OTS + H_2_O)	20.1	(10.4 + 20.1) × 5	≈100	95%	1.58 × 10^−3^ (85/85) 5.43 × 10^−7^ (RT)	1.31 × 10^−6^	×	10	720 h OK @ 85/85	×	[80]
		80	9		Alucone	1 nm	50 nm	×	×	7.10 × 10^−5^	9.94 × 10^−5^	×	1	×	×	[126]
		80	1 cyc *	PECVD	PP-He×ane (C_6_H_14_)	20	(0.11 + 20 nm) × 200	×	×	3.00 × 10^−4^	<20% degradation (10 k cycle)	(T)	5	44 h OK @85/85 (Ca test)	Same CE (20 dyads)	[127,128]
		70	60	Spin	Silamer	2000	(60 + 2000) × 3	281.6	90%	3.11 × 10^−6^	1.00 × 10^−1^	×	16.7	×	Same CE	[71]
		70	330 cyc (≈30 nm)		S-H Nano *	190	<700 nm (3.5 dyads)	×	85.80%	1.14 × 10^−4^	2.23 × 10^−4^ (1.5 dyads)	(T)	30	700 h @under ambient conditions	Same CE Inferior LT	[129]
		70				190		×	85.80%	5.43 × 10^−5^	6.97 × 10^−5^ (2.5 dyads)	(T)	30			
		70				190		×	85.80%	1.14 × 10^−5^	1.76 × 10^−5^ (3.5 dyads)	(T)	30			
		70	30	Spin/bar coating	S-H nanocomposite/hybrimer (neutral, 110 um)	120	480	×	88.20%	4.40 × 10^−5^	8.20 × 10^−5^	0.63%	10	720 h OK @30/90	Inferior CE	[130]
	Al_2_O_3_/ZnO	70	30 (3 nm/3 nm)	Spin	S-H nanocomposite	100	(30 + 100) × 3.5	×	>85%	7.87 × 10^−6^	7.78 × 10^−5^	0.63% (T)	10	×	Same CE better LT	[120]
		70				100		×			2.51 × 10^−5^	0.31% (T)	×	×		
		70				100		×			1.56 × 10^−5^	0.21 (T)	×	×		
		70	30			120	30/120/30/120/30	×	89.10%	1.91 × 10^−5^	4.05 × 10^−5^	0.21%	30	720 h OK @30/90	Same CE	[131]
	ZnO/Al_2_O_3_/MgO	70	50 (20/12/10)			140	50/140/50	117.2 (ZAM) 30.37 (ZAM/organic TFE)	91.40%	2.44 × 10^−6^	4.62 × 10^−6^	0.3% (T)	×	2000 h OK @RT		[132]
		70									8.20 × 10^−6^	0.62% (T)	×			
		70									2.65 × 10^−5^	0.89% (T)	×			
		70									9.78 × 10^−5^	1.04% (T)	7			
		70									4.39 × 10^−4^	1.25% (T)	6			
		70	30			1000	30/1000/30/1000/30	×	×	5.94 × 10^−5^	1.00 × 10^−4^	0.63%	10	240 h OK @60/90	×	[133]
ALD/PECVD	SiO_x_/AlO_x_	110	150 (100/50)	CVD	Perylene C	1000	1150	×	×	2.40 × 10^−5^	No crack	0.8% (T)	6.4	×	×	[92]
ALI	Al_2_O_3_	100	22	Spin	PI	20,000	20,022	×	×	1.00 × 10^−7^	No failure	×	1	1000 h OK @85/85 as substrate barrier	×	[134]
		140	7	Spin		10,000	10,007	×	×	1.40 × 10^−5^	No failure	×	1		Same CE	[135]
Dual-gun sputter	SiO_2_/Al_2_O_3_	RT	(10 + 10) ×24	N/D	UV resin	1000	1480	×	>82%	3.79 × 10^−5^	1.64 × 10^−3^	(T)	10	×	×	[121]
HW-CVD	SiN_×_:H	100	50	Spin	PMMA	300	1100	×	80%	9.20 × 10^−5^	1.10 × 10^−4^	0.89%	5	×	×	[136]
LBL/S-PEALD *	h-BN/Al_2_O_3_	80	20	N/A	N/A	N/A	N/A	×	>95%	1.80 × 10^−4^	<30% degradation	4%	3	×	×	[137]
LP PECVD	SiN_x_:H/ SiO_x_N_y_	120–130	400/4	Dip coating	ORMOSIL (PDMS/PUA/h-SiO_x_)	150	400/4/150/4/400/4/150	×	84%	9.20 × 10^−5^	5.00 × 10^−5^	×	2.5	×	Same CE Same LT	[77]
R-PEALD *	Al_2_O_3_	95	30	Inkjet	PMMA	2500	30/2500/30	×	95%	5.00 × 10^−5^	No failure	×	3.2	305 h OK @60/85	Better CE (30.2%)	[95]
PECVD	H: SiON	100	80	Spin	Acrylate-based polymer	1200	1280	×	87.80%	5.00 × 10^−5^	5 × 10^−5^ (10 k cycle)	×	1	720 h OK @RT	Higher CE	[83]

* cyc = cycle; * Dep. Temp. = deposition temperature; * iCVD = Initiated Chemical Vapor Deposition; * MLD = Molecular Layer Deposition; * S-PEALD = spatial PEALD; * R-PEALD = Remote PEALD; * S-H Nano = S-H Nanocomposite.

**Table 3:** A brief summary of WVTR, reliability, flexibility, advantages/disadvantages, cost, and maturity of different TFE strategies.

TFE Strategy	Achievable Level of WVTR (g m^−2^ day^−1^)	Achievable Reliability /h (Convert to 85 °C/85% RH)	Achievable Flexibility (@ Strain, Bending Radius)	Advantages	Disadvantages	Cost	Maturity
ALD/CVD inorganic laminate	~10^−2^ (maybe ~10^−4^ nowadays)	5.5	No data	Engineering available High D/R	Modest properties Little studied	Low	High
ALD monolayer	~10^−6^	205.5	No data	Engineering available	Modest properties Low D/R	Medium	High
ALD nanolaminate	~10^−5^	300.0	No data	Acceptable properties	Low D/R	Medium	High
ALD/iCVD nanolaminate	~10^−5^	720.0	Maintain barrier property @1.09% (T) R2.3	Excellent properties	Low D/R	High	Medium
ALD/MLD nanolaminate	~10^−7^	720.0	Little damage @0.72% R12	Excellent properties	Low D/R	High	Low
ALD/inkjet laminate	~10^−6^	70.9	No failure @R3.2	Engineering available	Modest properties Low D/R	Medium	High
ALD/plasma polymer nanolaminate	~10^−4^	44.0	<20% degradation (10 k cycle) @R5	Engineering available High flexibility	Modest properties Low D/R	Medium	Medium
ALD/S-H nanocomposite laminate	~10^−5^	63.2	Remain at the same WVTR level @0.63%, R10	Excellent properties	Low suitability for manufacturing	High	Medium
ALD/2D layer	~10^−4^	No data	Remain at the same WVTR level @1.25% (T), R6	Excellent flexibility	Low maturity Modest properties	High	Low
ALI	~10^−7^	1000.0	No failure @R1	Excellent properties	Low maturity Low D/R	High	Low
Others: H: SiON (low D/R PECVD)	~10^−5^	7.1	Remain at the same WVTR level @R1, 10 k cycle bending	Engineering available	Modest properties Low transparency(proposed)	Medium	Medium

## Data Availability

The original contributions presented in this study are included in the article. Further inquiries can be directed to the corresponding authors.

## Abbreviations

The following abbreviations are used in this manuscript.

LCDs	Liquid Crystal Displays
TFE	Thin-film encapsulation
GDBs	Gas diffusion barriers
WVTR	Water Vapor Transmission Rate
OTR	Oxygen Transmission Rate
OLEDs	Organic Light-Emitting Diodes
AMOLEDs	Active-Matrix Organic Light-Emitting Diodes
ALD	Atomic layer deposition
MLD	Molecular Layer Deposition
RA	Reliability
pp-HMDSO	Plasma polymer Hexamethyl disiloxane
TFTs	Thin-film transistors
COS	Crack onset strain
LED	Light-Emitting Diode
R & D	Research and design
IJP	Inkjet printing
PECVD	Plasma-enhanced chemical vapor deposition
TMA	Trimethylaluminum
PEALD	Plasma-enhanced atomic layer deposition
APP	Atmospheric Pressure Plasma
SiON	Silicon oxynitride
TEM	Transmission electron microscopy
iCVD	Initiated chemical vapor deposition
ALI	Atomic Layer Infiltration
PMMA	Polymethyl Methacrylate
CE	Current efficiency
DMD	Dielectric/metal/dielectric
PDMS	Polydimethylsiloxane
SALD	Spatial ALD
FCVA	Filtered Cathode Vacuum Arc
R2R	Roll to Roll
